# UV-Protective Compounds in Marine Organisms from the Southern Ocean

**DOI:** 10.3390/md16090336

**Published:** 2018-09-14

**Authors:** Laura Núñez-Pons, Conxita Avila, Giovanna Romano, Cinzia Verde, Daniela Giordano

**Affiliations:** 1 Department of Biology and Evolution of Marine Organisms (BEOM), Stazione Zoologica Anton Dohrn (SZN), 80121 Villa Comunale, Napoli, Italy; laura.nunezpons@szn.it (L.N.-P.); cinzia.verde@ibbr.cnr.it (C.V.); 2 Department of Evolutionary Biology, Ecology, and Environmental Sciences, and Biodiversity Research Institute (IrBIO), Faculty of Biology, University of Barcelona, Av. Diagonal 643, 08028 Barcelona, Catalonia, Spain; conxita.avila@ub.edu; 3 Department of Marine Biotechnology (Biotech), Stazione Zoologica Anton Dohrn (SZN), 80121 Villa Comunale, Napoli, Italy; giovanna.romano@szn.it; 4 Institute of Biosciences and BioResources (IBBR), CNR, Via Pietro Castellino 111, 80131 Napoli, Italy

**Keywords:** antarctica, UV radiation, ozone hole, climate change, marine organisms, sunscreen, UV-absorbing molecules, antioxidants, DNA repair, cosmeceuticals

## Abstract

Solar radiation represents a key abiotic factor in the evolution of life in the oceans. In general, marine, biota—particularly in euphotic and dysphotic zones—depends directly or indirectly on light, but ultraviolet radiation (UV-R) can damage vital molecular machineries. UV-R induces the formation of reactive oxygen species (ROS) and impairs intracellular structures and enzymatic reactions. It can also affect organismal physiologies and eventually alter trophic chains at the ecosystem level. In Antarctica, physical drivers, such as sunlight, sea-ice, seasonality and low temperature are particularly influencing as compared to other regions. The springtime ozone depletion over the Southern Ocean makes organisms be more vulnerable to UV-R. Nonetheless, Antarctic species seem to possess analogous UV photoprotection and repair mechanisms as those found in organisms from other latitudes. The lack of data on species-specific responses towards increased UV-B still limits the understanding about the ecological impact and the tolerance levels related to ozone depletion in this region. The photobiology of Antarctic biota is largely unknown, in spite of representing a highly promising reservoir in the discovery of novel cosmeceutical products. This review compiles the most relevant information on photoprotection and UV-repair processes described in organisms from the Southern Ocean, in the context of this unique marine polar environment.

## 1. Introduction

**Figure 1:**
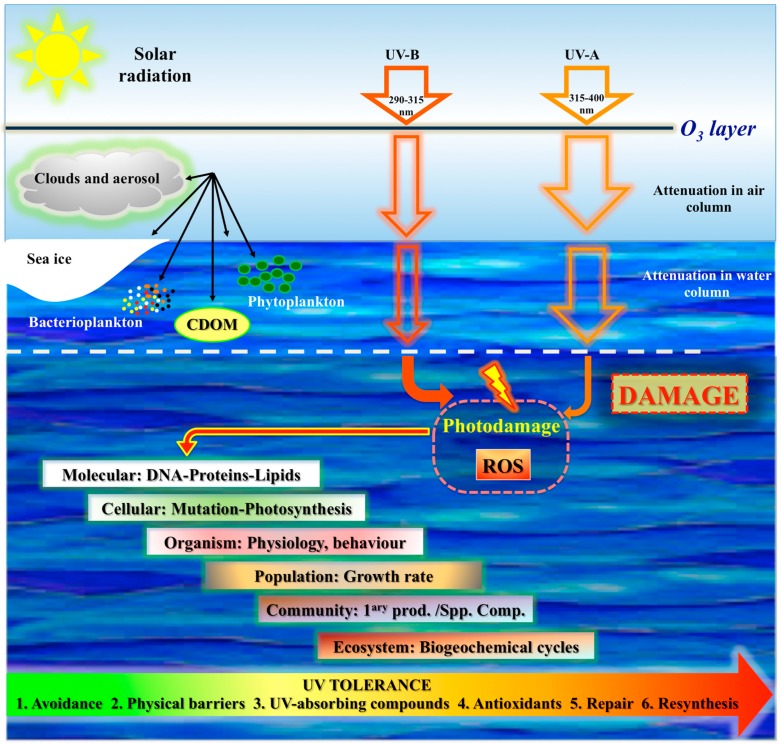
Negative effects of ultraviolet radiation (UV-R) on marine environment. Main factors involved in the attenuation of light through the air and water column and mechanisms of UV tolerance in biological systems by mitigating strategies and repair processes. UV-R may affect organisms through molecular and/or cellular damages, genetic mutations, or by causing disturbances at population and community levels, interfering with physiological functions (e.g., growth, reproduction and behaviour), and species interactions with effects on the ecosystem and biogeochemical cycles. ROS, reactive oxygen species; CDOM, coloured dissolved organic matter.

Ultraviolet radiation (UV-R) is one of the most critical abiotic factors for life on Earth. In spite of the beneficial effects, sunlight can also threaten living organisms, and excessive UV-R of certain wavelengths can promote damage in their molecular machineries. Such deleterious processes can alter marine ecosystems productivity, thus affecting species diversity, ecosystem stability, trophic interactions, and global biogeochemical cycles [1] (Figure 1).

The ozone layer in Earth’s atmosphere acts as a shield by absorbing biologically harmful solar UV-B (290–315 nm). However, each spring, large ozone holes develop over the Southern Hemisphere, increasing the amount of UV-B that reach the Antarctic marine environments [2]. The ecological consequences of springtime ozone depletion are directly correlated with the tolerance of species to UV-B via photoprotective strategies to minimize UV exposure, and repair mechanisms for correcting UV-B-induced damage (Figure 1).

Antarctic species have developed a variety of adaptive strategies to mitigate the effects of solar UV-B radiation, including avoidance mechanisms, synthesis of UV-absorbing substances, enzymatic and non-enzymatic quenching of reactive oxygen species (ROS), and the activation of DNA repair pathways. However, there is a critical lack of information about the UV-B photobiology of Southern Ocean biota before the occurrence of springtime ozone depletion, and about the ecological consequences after the first depletion events in the 1970s [3]. Current research on the UV photobiology of Antarctic marine organisms is still poor and characterized by an old literature on the theme. In this review, we describe the major characteristics of the Antarctic marine environment by outlining the principal geophysical properties influencing the Southern Ocean (e.g., currents, UV-R, photoperiod, seasonality, ozone depletion, temperature, sea ice dynamics and global climate change) in the context of marine photobiology. The most relevant available information on UV-protective strategies has been summarized, with particular emphasis on suncreen compounds, molecular quenching and photodamage-repairing mechanisms. Such processes have been further compared to those described at other latitudes, in order to identify analogies/differences in the chemical structure of the molecules involved, their function and specific concentration.

### 1.1. Antarctic Marine Environment

Antarctica is detached geographically from the other continents, and isolated oceanographically and thermally by currents (e.g., the Antarctic circumpolar current (ACC)) defining sub-zero temperatures. It was originally part of the supercontinent Gondwana, which originated from Pangaea and began to break up c. 135 million of years ago (mya), during the early Tertiary. Antarctica reached the current geographic position at the beginning of the Cenozoic, 65 mya [4]. The region currently comprises the major continental land, the Maritime Antarctic, the sub-Antarctic islands, and the southern cold-temperate islands. Over the Cenozoic, in the last 40 my, the Antarctic shelf experienced cyclical glaciations that led to isolation from other oceans, as well as the establishment of colder conditions, and to major episodes of extinction of marine fauna [5]. The earliest cold-climate marine faunas are thought to date back to latest Eocene—Oligocene (35 mya) [6]. Extensive and thick ice sheets began to form periodically every 1–3 my, after the middle Miocene. Climate records from ice and sediment cores indicate that over the past 800,000 years, polar regions have gone through eight glacial cycles [7]. The last glacial cycle is dated on ~120,000–110,000 years ago, and culminated approximately 15,000 years ago [8]. At that time, the ice sheet thickened to more or less its recent configuration. The progressive separation from other continental masses allowed the establishment of the ACC and, at its northern border, the Antarctic Polar Front (APF), creating the isolated and cold habitat we know today. A southward shift of the fronts of the ACC has been suggested as the key mechanism for some of the observed Southern Ocean warming [9].

Antarctica is renowned as being the driest, windiest and coldest continent, boasting the lowest recorded temperature on Earth, −89.2 °C [10]. In terms of water surface, the Southern Ocean (including the Weddell and Ross Seas) is the planet’s fourth largest ocean. Water temperatures range between +1.5 and −1.9 °C at the most northerly and southerly latitudes, respectively [11]. There is little variation in temperature during seasons or as function of depth, because of the presence of the ACC and APF [12,13]. Along the APF, the surface layers of the north-moving Antarctic waters sink beneath the less cold and less dense sub-Antarctic waters, generating almost permanent turbulence [14]. Here the Ocean plays an important role in the global carbon cycle being responsible for ~20% of carbon dioxide (CO_2_) drawdown [10]. The deep water south of the APF, a roughly circular oceanic system extending to 2000 m in depth, brings to the surface dissolved nutrients and CO_2_, and then releases CO_2_ to the atmosphere. In contrast, water north of the APF takes up CO_2_ from the atmosphere thus making the Southern Ocean both a source and a sink for atmospheric CO_2_.

APF acts as a barrier for migration of marine organisms between the cooler Antarctic and the lower warmer latitudes [14]. Moreover, the cooling process over the last ~5 my, characterized by cyclical freezing and warming events accompanied by advances and retreats of the continental ice sheet, introduced new niches for faunal radiation [15,16]. More than 9700 species, mostly benthic, have been described from the Southern Ocean. The number of known species has significantly increased in the last years thanks to initiatives, such as the Census of Antarctic Marine Life [17]. Marine organisms thriving in the Southern Ocean are exposed to extreme conditions of isolation, harsh climate, low stable water temperature and viscosity, deep continental shelf and disturbance from scouring by icebergs [18,19]. Several studies revealed that despite some taxonomic connectivity that remains with South America through the Scotia Arc (which acted as biogeographic bridge between Antarctica and the Magellanic region [20]), the current benthic marine invertebrate fauna is largely ancient and endemic [21]. As such, the biota has had chances to co-adapt to this unique severe environment [22].

### 1.2. UV Radiation, Penetration, Photoperiod

Solar UV-R, the portion of the electromagnetic spectrum between X rays and visible light, was an important factor in driving the evolution and ecology of the biosphere until the development of the ozone layer and photoprotective mechanisms. UV wavelengths range from below 200 nm to 400 nm and are divided in vacuum UV (less than 200 nm), UV-C (200–290 nm), UV-B (290–315 nm), and UV-A (315–400 nm). The energy associated with a photon is inversely proportional to its wavelength; the higher is the energy, the greater is the capacity of UV-R to cause damage [23]. UV-R at the Earth’s surface varies with the season, time of day, latitude, and altitude. The local incidence of UV is determined by the total ozone column, cloudiness, ground reflectivity (i.e., the albedo), and local aerosols. In Antarctica, aerosols are almost absent, and the role of clouds is less important than in other parts of the planet. Consequently, surface UV in Antarctica is mostly driven by ozone and albedo [24].

The effects of UV-R on biogeochemical reactions in the sea (that is, mainly dissolved organic matter (DOM), bacterio- and phytoplankton) depend on the attenuation of the water column, that in turn depends on the optical properties of seawater itself, dissolved material, concentration of phytoplankton and suspended particles [1]. Colored or chromophoric dissolved organic matter (CDOM), yellow substance, or gelbstoff, is a result of tannin-stained decaying detritus from macroalgae and plankton, due to microbial degradation. CDOM can strongly absorb short wavelength light (from blue to ultraviolet), controlling the optical characteristics of freshwater and coastal habitats. This leads to the attenuation of both, photosynthetically active radiation (PAR, 400–700 nm) and UV-R, thus reducing UV exposure of organisms in the water. Absorption of solar UV-R causes the bleaching—photodegradation—of CDOM, reducing its optical density and absorptive properties. This not only increases the transmission of radiation in the water column, but it also originates low-molecular-weight organic compounds and ROS, which can be deleterious to marine organisms [1]. Even though concentrations are highly variable, coastal water and estuaries are usually richer in CDOM than the open ocean, where UV-R can penetrate deeper. Organisms living in oceanic waters may hence be adapted to higher and stable levels of UV-R [25]. Oligotrophic tropical waters (e.g., coral reefs) are in general more transparent to UV-R than temperate waters, because the optical properties depend on the water itself and not on dissolved constituents [26] (and references herein).

Antarctic waters are characterized by a low attenuation of UV-R, particularly during episodes of ozone hole. Here, the surface incidence can increase by 35% during spring time ozone depletion events [25]. In a comparative study around the Southern Ocean, Fildes Bay (South Shetland Is.) exhibited by far the highest UV penetration, recording 11 m for UV-B (313 nm) and 27 m for UV-A (395 nm), versus 2 and 4 m respectively in a sub-Antarctic Chilean fjord [27]. Smith et al. [28] found that in the ozone hole at Bellingshausen Sea effective UVR penetration could increase by 7 m and could be detected down to 60–70 m depth. Optical properties in Antarctic waters are further regulated by snow, ice cover and particulate material from runoff during melting [27]. Although annual sea ice may be considered a physical and protective barrier to UV-R transmission, it has been demonstrated that UV-B is transmitted through the Austral spring annual ice of McMurdo Sound, when the ozone hole occurs, causing mortality and DNA damage in the embryos of the sea urchin *Sterechinus neumayeri*. Higher mortality and DNA damage occur at 1 m below the ice with respect to 3 m and 5 m [29].

Seasonal change in day length (photoperiod) is widely used by organisms to regulate temporal patterns of development and behavior. The Antarctic continent experiences 24 h of continuous daylight at the summer solstice in December and 24 h of dark at the winter solstice in June. Therefore, at high latitudes, sunlight is strongly seasonal, and ice-free days around the summer solstice receive orders of magnitude more light than those in winter [30]. To remain viable under extended dark conditions, cells must retain membrane, organelle and DNA integrity, as damage is constantly occurring, due to metabolic heat and oxidation. Low temperatures in some way reduce such processes, but still polar phototrophic cells rely on different strategies to survive long periods of darkness. These include production of cysts or dormant stages, switch to heterotrophy (mixitrophic) or reduction of metabolic rates and rely on energy storage products. Spore production is quite uncommon in Antarctic phytoplankton, and has been observed only in some diatom species, and a few dinoflagellates. Facultative heterotrophy, in lieu, is more widespread. During the dark season pigments and UV-absorbing products decline to minimum levels, and upon the appearance of the first rays of light, most phytoplankton taxa are able to resume photosynthesis in hours. Furthermore, one seaweed species was able to reactivate within 24 h after a six-month period of total darkness (reviewed in Reference [31]). Despite lower temperatures and stronger seasonality, the overall photosynthetic efficiencies (the proportion of captured photons channeled to photosynthesis) are similar between higher and lower latitude ecosystems thus implying that the polar species (phytoplankton) are shade adapted [32].

### 1.3. Sea Ice Dynamics

Antarctic ice is one of the main features of the Southern Polar continent, covering ~99.6% of its land area and surrounding seas. This ice sheet extends an average of 30 × 10^6^ km^3^ and represents 70% of the Earth’s freshwater. There are several typologies of ice, which prompt impacts to marine biota living on or in the ice, and to subtidal and underlying ecosystems. Freshwater ice derives from glaciers, it can extend up to a kilometer thick, and form the ice shelves attached to the land, and freely floating icebergs when large masses of ice detach [33]. The direct effects of iceberg scouring, and anchor ice, are major factors of physical disruption for benthic communities in Antarctic seafloors [34]. Sea ice, instead, is frozen seawater a few meters thick, and similarly subdivided into fast ice (attached to land) and ice floes (non-attached) [33]. In the Southern Ocean the ice cover is highly seasonal, spreading each winter far northward (to approximately 60° S) and experiencing a retreat almost to the coastline in the summer [35]. Antarctic sea ice mediates physical disturbance to the benthos. On the one hand it prevents drifting icebergs from scouring the seabed; and on the other it forms a barrier between the water column and the atmosphere, restricting wind-induced turbulence and water-column turnover [36]. These reduced land–ocean–atmosphere interactions interfere with the supply of nutrients (particulaly iron) to the marine environments [37]. Algae and particles trapped within sea ice caps seasonally constitute the basis of the Antarctic marine food chain when the packs melt, and phytoplankton blooms and organic matter reach the benthos [38,39]. Regarding the effects on sunlight incidence, ice sheets strongly modify the radiation budget and energy balance on the ocean surface by reflecting light (albedo). The regular albedo without snow is 6–7%, but it can exceed 85% in the presence of sea ice [40]. In underwater ecosystems, sea ice significantly attenuates solar irradiance [40] while protecting from UV wavelengths [41]. For instance, the Weddell Sea pack ice in the austral spring (September) showed an almost dark under-ice light regime with light transmittances bellow 0.1% [42]. For all the mentioned, the ecology and productivity in the Southern Ocean are strongly influenced by the sea-ice cover and its periodicity [43,44]. Each year Antarctic ecosystems go through a sort of lethargy marked by long dark cold winters, and reactivate in the summer upon the return of sunlight and the melting of the ice. These cyclic events influence all marine biota, but in particular photoautrotrophs and organisms depending on these for nutrition or light protection [43,45]. Contradicting most global climate models (including recent reports of Arctic ice declines [46]), Antarctic sea ice extent increased in the last decades [47]. These episodes of sea-ice expansion increased surface albedo, reduced ventilation, and enhanced CO_2_ sequestration to the deep ocean [48]. Nonetheless, since 2016/17 unprecedented springtime retreats in the Antarctic ice packs [49] highlighed the possibility of a switch to future declines in sea ice extent [39]. Reductions in the fraction of ice and snow cover will definitely influence the exposure of marine ecosystems to solar UV-R, and consequently the biology of photosynthetic organisms at the base of the food web, invertebrates and large predators along the ecological web (reviewed in References [35,50]).

### 1.4. The Ozone Hole and the Impacts of a Changing Environment

Although there are some contrasting opinions, it is now well-accepted that in early atmosphere there was no oxygen (O_2_) and therefore no UV protective ozone layer [51,52]. In these conditions, cells may have been confined to dimly lit regions of the oceans. The evolution of photosynthetic organisms as cyanobacteria resulted in oxygenation of the atmosphere and the formation of the ozone layer. When O_2_ accumulated in the upper atmosphere, it was photochemically transformed to ozone (O_3_), filtering out the shortest wavelengths of UV-R and thus changing the evolution of life on Earth [26,51,52]. The stratospheric ozone layer screens harmful UV-R from the Earth’s surface thus protecting against adverse effects on cells. The shortest and most damaging wavelengths UV-C are strongly absorbed in the upper atmosphere not reaching the stratospheric ozone layer. UV-B wavelengths are absorbed by ozone, which modify both the spectral quality and the intensity of UV-B radiation, allowing only a small amount to reach the Earth’s surface. The longest wavelengths UV-A are not absorbed by ozone layer and thus are not dependent by ozone concentrations [3].

In the early 1970s, scientists recognized that human actions producing chlorofluorocarbons (CFCs) could deplete the protective layer of ozone [53], destroying virtually all ozone between heights of 14 and 22 km over Antarctica [46], especially within the south polar vortex (persistent, large-scale cyclone) where temperatures are coldest. UV-R breaks down CFCs producing significant amounts of chlorine radicals that in turn react with ozone, catalyzing its destruction [54].

Currently, the ozone layer is diminished, particularly over the Southern Hemisphere and occasionally develops over the Arctic [55]. The “ozone hole” over most of Antarctica has grown in size (up to 27 million km^2^ in 2006, which is nearly twice the area of the Antarctic continent) and duration (from August through early December) over the past decades. At present (November 2017), the area is between 15–18 million km^2^ [56].

Although the photodissociation of CFCs can occur all across the Earth, the cold dark and long Antarctic winter favours, more than in any other part, the accumulation of chlorine in the Southern Hemisphere [57,58]. This leads to the destruction of ozone molecules in the spring, when the sunlight returns to the Austral latitudes from September to October [59].

The depletion in polar regions is larger than at lower latitudes, yet it accounts for only about 13% of Earth’s surface. Ozone depletion also develops at latitudes between the equator and polar regions: total ozone averaged for 2008–2012 has been about 3.5% lower in northern midlatitudes (35° N–60° N) and about 6% lower at southern midlatitudes (35° S–60° S) [60]. In the tropics (20° N–20° S latitude), total ozone has been only weakly affected by chemical ozone depletion, because of the lower conversion of ozone depleting substances (ODSs) to reactive halogen gases [60]. In tropical regions, however, coral reefs are currently experiencing the highest irradiance of UV-R at sea level on Earth even in comparison with the Antarctic region [26].

Thanks to the Montreal Protocol (1987), the chemicals responsible for the depletion of the ozone layer are now largely regulated. The tendency is to reduce the production and use of CFCs and regulate the emission of ODSs, including greenhouse gases (GHG) [61]. The Protocol has led to a successfully reduction of concentration of GHG in the atmosphere, this way mitigating the climate-forcing across the globe [62]. Recently, Kuttippurath and Nair [63] reported the first practical results from the Montreal Protocol. In fact, Antarctic ozone depletion has started to recover in both spring and summer thanks to the reductions in global ODS emissions and continuing recovery is expected to occur. However, changes in ozone levels are not only due to the halocarbons, since significant changes had already taken place, due to other source gases (e.g., N_2_O, CO_2_, CH_4_). Recently, the interest in the long-term recovery of the ozone layer refocused the attention on the effects of these gases on global mean ozone levels [64].

Interaction between climate change, ozone, and UV-R may be of considerable importance on the fate of marine organisms and entire ecosystems, and must be studied in a multifactorial manner in order to understand the impacts on our future oceans [65]. In the last 30 years, the Southern Ocean has changed notoriously [10,49], with profound implications in marine ecosystems, although some effects seem to be more regionally specific [37].

Ozone depletion is one of the major drivers of climate change in the Southern Hemisphere [66]. Higher air temperatures and incoming solar radiation are increasing the surface water temperatures of lakes and oceans, reducing annual snow and ice cover and, thus, increasing exposure to UV-R. As a consequence, warmer oceans are changing the composition of many marine ecosystems and their services and functions [65]. In the tropics and also the Mediterranean, temperature raises of the seawater have devastating effects, causing bleaching, disease and stress-related outbreaks on marine organisms [67,68,69]. Biotic networks in high latitudes seem to be considerably vulnerable [70,71], given that polar species are in general stenothermal and therefore, less capable of enduring temperature shifts [72,73]. The warming of the water along the Antarctic Peninsula has been five times faster than the global average over the past 50 years [65], with an increase of ~0.5 °C/decade since 1950 [74]. Reduction of the seasonal sea-ice, increased ocean temperatures both in the Weddell and Bellingshausen seas, regional retreat of glaciers, disintegration of floating ice shelves, expansion of terrestrial flora, and permafrost degradation are effects of the fast climate changes observed in the Antarctic Peninsula [49,75]. Finally, higher atmospheric CO_2_ concentration induces ocean acidification, alters seawater chemistry impairing the formation of UV-absorbing exoskeletons in many marine organisms, including phytoplankton, macroalgae, and animals, such as molluscs and corals. As an example, the shells of pteropods, key species in the food web, are already dissolving in areas of the Southern Ocean surrounding Antarctica [76].

## 2. Effects of Light in Marine Organisms

Life on Earth relies on sunlight [77]. The infrared rays of longer wavelengths (700 nm to 1 mm) are responsible for warming contributing to the benevolent temperatures of our planet; whereas the visible spectrum (400–700 nm, visible for human eye) supports the sense of sight. Remarkably, the visible light is also essential for photosynthesis, the process whereby autotrophic solar-powered organisms that are at the basis of most food networks derive their energy from photons. However, on the other side of the spectrum below 400 nm, UV sunlight exerts mostly deleterious effects on biological systems [77,78].

### 2.1. Beneficial Effects of Light

The sea covers about 71% of the planet’s surface and contributes to about one third of the global productivity. In marine systems above the aphotic zone, UV-R penetrates deeply and biota at all trophic levels become potentially exposed to UV-R [79]. The principal marine primary producers comprise planktonic diatoms, dinoflagellates, coccolithophorids, silicoflagellates, and blue-green and other bacteria, while benthic phototrophs include micro- and macroalgae, higher plants, and symbiotic producers, such as zooxanthellae in corals. All these organisms live in the euphotic zone to remain photosynthetically active. Zooplankton, herbivores, and other heterotrophs, in turn, largely depend on those photoautotrophs as their primary source of food. Marine biota depends directly or indirectly on light, for a number of biological processes [80,81]. Besides photosynthesis-related effects, there are other beneficial processes powered by light. For instance, beyond the photoautotrophic nourishment zooxanthellae provide to their coral hosts, these symbiotic dinoflagellates can further potentiate calcification and lipogenesis processes in the presence of light [82]. UV-R has also been shown to be necessary for spicule formation in some gorgonians, as colonies maintained in the absence of UV-R had significantly more “irregular” spicules when compared to colonies grown in the presence of UV-R [83]. For swimming organisms, the capacity of phototaxis may allow them to control their position in the water column, while avoiding excess of radiation [84]. UV photoreceptors have been described in bacteria, cyanobacteria, and algae, as well as in protozoans, annelids, cnidarians, molluscs, crustaceans, and fish, suggesting that UV vision may be relevant in aquatic systems [79,85]. UV photoreceptors may be used for navigation, communication, enhanced foraging, and possibly for UV-R avoidance. For instance, in the Antarctic krill *Euphausia superba*, a complex photoreception system, composed of different opsin photopigments, enables to respond to the daily and seasonal changes in light, moving downward during the day and upward during the night within the top 200 m of the water column [86]. Both negative phototactic behaviors and UV vision, suggest that UV-R may influence behaviour, migration and abundance patterns, as well as predator-prey and intraspecific interactions in marine environment [79,85]. UV-R may also play an essential role in the ecology of several infectious diseases of aquatic organisms, particularly when there is a pronounced difference in the UV tolerance of the host and the pathogen or parasite [65]. Simultaneously, solar radiation is very effective at reducing viral infections in some organisms, including fish viruses and harmful algal blooms, as well as some trematode worms infections [65].

Light is essential for the synthesis of vitamin D (calciferol) in most organisms, which has a significant role in calcium homeostasis, inmune system, and metabolism [87]. Its precursor, 7-dehydrocholesterol reacts with UV-B light at wavelengths between 270 and 300 nm, with peak synthesis occurrying between 295 and 297 nm [88].

Seasonal cycles in many organims may also be controlled by light, since light actually initiates different kinds of cycles as it increases in spring [80]. Duration and extent of the effects are variable. Vertical migrations, for example, happen usually within a day (diurnal cycles), while horizontal migrations are seasonal or annual. Intertidal organims also use light to adjust their optimum position relative to tidal height [82]. There are different types of mechanisms based on light to synchronize individuals of a given species, or to regulate a large number of activities [77]. Photokinesis, photoperiodicity, photosensibilization are among these mechanisms. Furthermore, circadian rhythms, which are endogenous, are also often regulated by light, and may also have intermediate controls, such as hormonal regulation [77]. Somatic growth and reproduction are usually coupled with seasonal cycles [82]. Synchronization may be also very useful for reproduction and for survival of the offspring, and many sponges, corals, and echinoderms, for example, spawn in a coordinated way related to light [82].

### 2.2. Negative Effects of Light

The ozone layer is continuously depleting and, as a consequence, there is an increase in the incidence of UV-R reaching the Earth’s biota that can be then absorbed by selected biomolecules (e.g., DNA, proteins porphyrins, carotenoids, steroids, quinones), causing direct damage in both plants and animals, and sunburn in humans [77,78]. The highly energetic wavelengths when absorbed by DNA [89] can cause damage (e.g., cyclobutane pyrimidine dimers, CPDs), and mutations either directly by absorption or indirectly due to the production of ROS [90]. Indeed, a routine way in which UV-R can harm marine organisms is via photochemical reactions and generation of ROS. Reduced O_2_ intermediates, such as hydrogen peroxide (H_2_O_2_), superoxide radicals (O_2_^−^•), hydroxyl radicals (•OH) and singlet oxygen (^1^O_2_) are produced as a result of electronic excitation after UV-R absorption and reduction of molecular O_2_. Most of the production of ROS involves the activation of intermediate molecules in cells (e.g., aromatic amino acids), which absorb UV-R, and enter into an excited state leading to the production of extremely reactive hydroxyl radicals in an iron-catalyzed Fenton reaction. UV-A-generated ROS trigger several toxic responses in organisms, including impair of DNA, enzymes, membrane proteins and lipids (especially those containing polyunsaturated fatty acids), as well as photooxidative stress of photosystem components in photoautotrophs (see in more detail in sections below; [91,92]).

In organisms that perform oxygenic photosynthesis, an excess of UV-B light can interfere with the thylakoid photochemistry, leading to a decrease in O_2_, electron transport, Rubisco activity and CO_2_ fixation rates [93,94]. Such processes consequently lead to photoinhibition or photoinactivation, bringing repercussions to the first levels of foodwebs [81,95]. Similarly, corals are affected by strong UV light if this impairs the photosynthetic capacity of their symbiotic algae resulting in reduced carbon supply. This consequently leads to decreased growth and calcification, reduced photosynthesis and changes in respiration, DNA damage, oxidative stress and eventual mortality, as well as adverse effects on reproduction, larval development, and settlement [26].

The tolerance levels and responsive behaviours to UV stress can be different from species to species. UV-R can induce relevant changes in population compositions and trophodynamics, shifting communities towards more UV-tolerant species [79]. UV-R may affect organisms directly by producing cellular and/or tissue damages, or it may also affect organisms indirectly by constraining them to suboptimal habitats where temperature and food abundance may be low and the predation risk high [79]. Elevated UV-B radiation affects the survival of phytoplankton by decreasing their motility and inhibiting their phototactic and photophobic responses [78,96]. For zooplancton, UV-B irradiation may cause irrevesible damage and/or death, and decrease the fecundity of survivors [97,98,99]. These phenomena could eventually lead to a decrease in invertebrate and fish populations that feed on zooplankton, and be transmitted along the trophic chain, eventually affecting humans. Juvenile polychaetes showed reduced growth and development of tentacles when fed detritus derived from diatoms previously exposed to artificial UV-B radiation versus diatoms that were not pre-exposed to UV-B radiation [100]. Apoptosis is promoted due to UV-R in developing sea urchin embryos [101]. Algae and seagrasses experience physiological, biochemical, morphological, and anatomical changes towards UV-R, with deleterious effects on growth, reducing leaf size and limiting the area available for energy capture [78]. The vertical distribution of seaweeds in their ecosystem is indeed strongly determined by solar UV-R. Specially developing brown and red algae are particularly sensitive [102]. Increased solar UV-R can also reduce recruitment and impact heterotrophic species, as well as primary producers [103].

Very few studies exist on the repercussions of UV-R in Antarctic marine communites. Some examples include the study on the viability of bacterioplankton, which was shown to decrease with depth, with no significant inhibition at 9.5 m depth [104]. UV-B radiation inhibits the growth of Antarctic sea ice microalgae *Chlamydomonas* sp. ICE-L, especially at high intensity [105]. In some macroalgae UV-R lowered germination, showing that at unicellular life stage there was a strong species-specific susceptibility to changes in the UV-R [106]. This is important in determining the upper distribution limit of Antarctic seaweeds, which affects the community structure. Propagules of three Antarctic intertidal macroalgal species *Adenocystis utricularis*, *Monostroma hariotii* and *Porphyra endiviifolium*, particurarly sensitive to environmental perturbations, showed no long-lasting negative effects to UV demonstrating the possession of good repair and protective mechanisms, necessary condition for the ecological success in intertidal habitats [107]. UV-B-driven DNA damage and mortality in Antarctic sea urchin embryos has been found to vary from year to year, depending on the thickness of the sea ice and total column ozone [29].

## 3. UV Photoprotection in Marine Organisms: Antarctic and Non-Antarctic Strategies

Marine organisms have developed physiological and biochemical traits to cope with UV. The choice of habitat is the most effective defence, and consists of avoidance mechanisms, such as cyclic migrating behaviours from high to low UV-R levels in a diel or seasonal manner, or translocation to shaded or deeper zones along the water column. Many cyanobacterial communities in Antarctica live in dim-light environments, such as within or beneath rocks, in permanently ice-covered lakes, beneath the surface of the soil or at the base of the plants within moss banks [108]. In the Antarctic cyanobacteria *Oscillatoria* sp. vertical migration of the microbial mat reduces the exposition to UV [109], whereas vertical mixing of the water column provides similar effects in planktonic organisms [96].

For organisms living exposed to sunlight, mechanisms for minimizing UV damage include: (1) Screening mechanisms for reducing UV exposure by physical barriers or chemical barriers with UV-absorbing compounds; (2) quenching mechanisms by non-enzymatic (carotenoids, α-tocopherol, ascorbic acid, glutathione) and enzymatic antioxidants, such as superoxide dismutase (SOD), catalase (CAT), glutathione peroxidase and other enzymes that can neutralize effects of radicals produced by UV photochemical reactions; and (3) repair mechanisms to deal with UV-induced damage that occur in DNA, proteins and lipids.

### 3.1. Physical Structures for Light Avoidance

Physical barriers consist of morphological or structural features that represent the first line of defence for preventing physical external injuries (e.g., from predators and enemies’ attacks), such as the calcified shells of gastropods and crustaceans or spines of echinoderms. Some structural devices also protect from harmful light forming an opaque barrier between the UV rays of the Sun and the body [3]. For instance, ostracods living in shallow benthic habitats possess shells that block 60–80% of UV-R, whereas the exoskeleton of planktonic *Daphnia* can block up to 35% [110]. Embryos of intertidal gastropods are also protected by egg capsules [111], even if they combine presence of photoprotective molecules [112]. Similarly, embryonic developing modes in limpets, *Crepipatella* spp., rely on sunscreen products prior to developing a protective shell, and adult females can transfer them to capsule walls, embryos and nurse eggs, according to their type of embryonic development [113]. The spicules, at least in the colonial didemnid ascidian *Didemnum mole*, are potentially related to UV-R photoprotection in shallow water colonies [114]. The tunics of some ascidians are pigmented and can absorb UV-R providing photoprotection [115].

A number of marine organisms produce copious mucous secretions that provide physical protection against sediments, desiccation and predation and might also decrease the damaging effects of UV-R. This UV-R-screening capacity may be attained by the optical properties of the mucous itself, but also because of the presence of UV-R-absorbing compounds that are excreted within mucous, e.g., very common in corals [26,116,117,118].

Cyanobacteria synthesize extracellular polysaccharides (EPS), high-molecular-mass heteropolysaccharides, which provide a protective matrix from UV stress [119]. In Antarctic cyanobacterial mats from the McMurdo ice shelf, EPS were found to be involved in matrix formation and also in the attachment to the substrate [120]. Other Antarctic marine bacteria produce EPS, which have proved to serve as ligands for trace metal nutrients and cryoprotection at low temperature and high salinity [121,122], but they may be as well involved in UV protection even if this function has not been reported yet.

Some EPS from marine bacteria are already commercially available in cosmetics under the name of Abyssine^®^ for soothing and reducing irritation of sensitive skin against chemical, mechanical and UV-B aggression [123].

In large-celled species, including some microalgae, plant pollen and spores, sporopollenin, biopolymer of variable composition that functions as antimicrobial agent, and confers rigidness to the cell wall [124] may further protect from UV-R by increasing the optical density. It was reported that species of microalgae, that were highly tolerant to UV-R had substantial amounts of sporopollenin, whereas species containing little or no sporopollenin were highly UV-R susceptible [124]. In the pollen and spores of Antarctic plants, sporopollenin is considered as bio-indicator of solar UV-B and a valuable archive for the reconstruction of past solar UV-B [125].

Some unicellular organisms display transitory mechanisms to screen UV. Symbiotic *Symbiodinium* algae, for instance, can produce multiple layered cell walls when exposed to UV-R, which disappear when returned to lower light conditions [126]. Moreover, the dinoflagellate *Scrippsiella sweeneyae* can increase in cytoplasm volume when exposed to UV-R, possibly to lengthen the path that damaging photons have to travel to reach internal components, e.g., DNA [127]. Actually, the amount of DNA damage in Antarctic phytoplankton is inversely correlated with the cellular size; smaller cells with higher concentrations of photoproducts in their DNA are more sensitive to UV-B than larger phytoplankton species and undergo greater damage [96].

### 3.2. UV-Absorbing Substances (Sunscreen)

The best known photoprotective response in marine organisms is the production or accumulation of UV-absorbing compounds, including mycosporine-like amino acids (MAAs) as the most common compounds with such properties, but also others, such as scytonemin, 3-hydroxykynurenine, melanin, various secondary metabolites and fluorescent pigments [118,128,129,130,131].

#### 3.2.1. Mycosporine-Like Amino Acids (MAAs)

##### Structure, Biosynthetic Pathways of MAAs and Their Regulation

**Figure 2:**
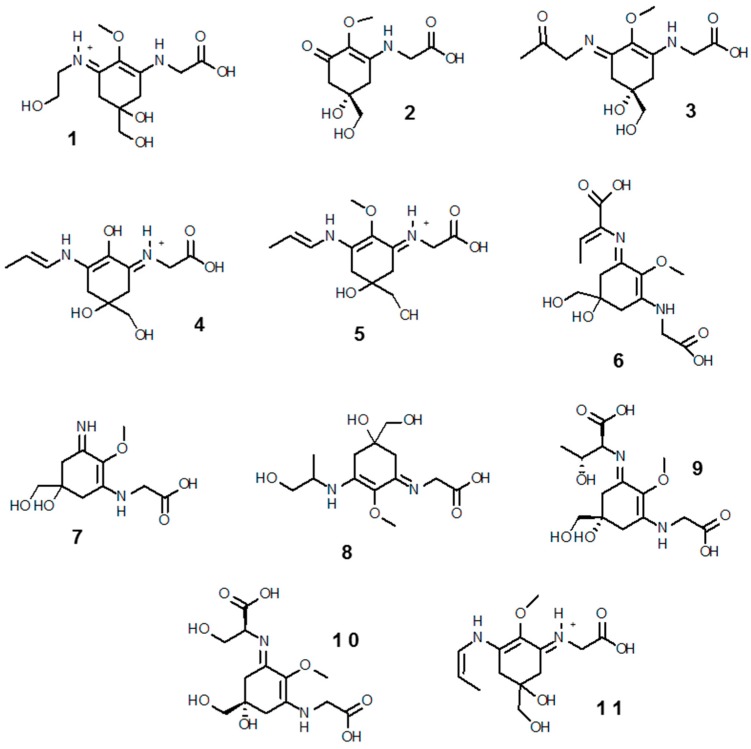
Mycosporine-like amino acids (MAAs) found in Antarctic marine organisms: (**1**) Asterina-330; (**2**) mycosporine-glycine; (**3**) mycosporine-2-glycine; (**4**) mycosporine-glycine-valine; (**5**) palythene; (**6**) palythenic acid; (**7**) palythine; (**8**) palythinol; (**9**) porphyra-334; (**10**) shinorine; (**11**) usujirene.

MAAs are small (<400 Da) intracellular, colorless water-soluble secondary metabolites of low molecular weight, commonly found in marine environments. Their name comes for being imino carbonyl derivatives of mycosporines—a group of compounds first identified in the mycelia of fungi as compound P310, and hypothesized to act as photoprotectants during sporogenesis [132]. Novel molecular species (characterized solely by their maximal light absorbance) are constantly being discovered; to date ~30 MAAs have been resolved, of which 11 (**1**–**11**), shown in Figure 2, have been reported in the Southern Ocean (see below). Their designation consists on the name and value of absorbance (e.g., euhalothece-362; Reference [133]). Characterization of MAAs should be treated with caution, as often it has been done by indirect comparisons on co-chromatography with sub-standards and/or with published UV spectral data and HPLC retention times [45,134,135]. MAAs are enamino ketones that contain a central aromatic aminocyclohexenimine or amonicyclohexenone ring and a wide variety of substitutions. These aromatic cyclohexenimine or cyclohexenone structures are responsible for the light absorption properties and accommodation of free radicals related to enhanced solar UV-R (see below; Reference [136]). The core cyclohexenone unit is derived from the first steps of the shikimate pathway, where 4-deoxygadusol—a strong antioxidant—is a direct precursor [137]. The most common MAAs contain a glycine moiety on the C3 of the cyclohexenimine ring and a second amino acid (porphyra-334 (**9**), shinorine (**10**), mycosporine-2-glycine (**3**), mycosporine-glycine-glutamic acid), amino alcohol (palythinol (**8**), asterina-330 (**1**)) or an enaminone system (palythene (**5**), usujirene (**11**)) linked to the C1. Eventually, further substitutions added as as side chains can take place [138].

The shikimic pathway is a metabolic route used by bacteria, cyanobacteria, fungi, algae, some protozoan parasites and plants for the biosynthesis of folates and aromatic amino acids (phenylalanine, tyrosine, and tryptophan), as well as of higher plant photoprotectants (i.e., flavonoids; [137]). It involves several enzymes that have been recently reported also in metazoan organisms; however, these are believed to still derive mostly from associated microbiota (bacteria, dinoflagellates). Indeed, MAAs are commonly described as “microbial sunscreens” [139,140]. Interestingly, Osborn et al. (2015) [141] recently reported that fish can de novo produce on their own gadusol—an antioxidant and UV-protective compound related to MAAs synthesis, and that analogous pathways are shared in amphibians, reptiles, and birds.

In many microorganisms MAAs are found in the cytoplasm where they are produced [142], yet in some cases, as in the cyanobacterium *Nostoc commune*, they can be excreted and extracellularly accumulated, showing more effective protection against UV-R [143,144]. The induction of MAAs synthesis has been proposed to be triggered via two disparate signal transduction pathways: One activated by UV-R and the other related to salt stress [145]. A UV-B specific photoreceptor called pterin involved in MAA production has been described in cyanobacteria [146]. In the red alga *Chondrus crispus* a receptor with absorbance peaks at 320, 340 and 400 nm (UV-A) has been linked to the formation of shinorine (**10**) [147]. Light-induced synthesis of MAAs can be as rapid as several hours in organisms like dinoflagellates *Alexandrium excavatum* and *Prorocentrum micans*, that experience rapid light changes during vertical migration [148], but it is usually slower [149,150,151].

Blue light in the PAR spectrum and UV-A promotes the production of MAAs in Antarctic diatoms [152,153,154], whereas corals require a combination of UV-B, UV-A and PAR for the synthesis of MAAs [155,156].

Dietary/trophic accumulation and microbial symbiont translocation are instead the via for the obtention of MAAs in many marine animals (e.g., References [84,149,157,158,159,160,161]).

##### Biological Functions of MAAs

MAAs have the capability to absorb light between 309 and 362 nm and dissipate radiation as heat without producing ROS [134]. The presence of a suite of MAAs extends the photoprotective potential and allows the harboring organisms thrive across a broader light spectrum [45,162,163].

The photoprotective role of MAAs has been demonstrated in several assays where extracts enriched in these UV-R absorbing substances significantly reduced the production of deleterious thymine photodimers by direct molecule-to-molecule energy transfer process [164]. MAAs are multi-functional sunscreens that exhibit photostability and resistance to several abiotic stressors [45]. Besides protecting cells from mutation caused by UV-R and free radicals, they are also effective antioxidant molecules via stabilizing free radicals within their ring structure scavenging ROS [133]. Mycosporine-glycine (**2**) in particular, can display several antioxidative properties, and this probably explains why it is the most frequently observed (and often abundant) MAA, in tropical environments, especially in cnidarians [165,166,167,168,169,170]. Mycosporine-glycine (**2**) yields rapid protection against oxidative stress, even prior the intervention of antioxidant enzymes [171] and this is achieved by scavenging free radicals [172] and quenching singlet O_2_ [173].

It has beed proposed that MAAs can further act as osmolytes and boost cellular tolerance to desiccation, salt, and heat stress [142]. Even if mycosporine-glycine (**2**) and shinorine (**10**) can be induced by salt stress, their role as osmolytes seems to be still ambiguous [145]. There are studies demonstrating their efficacy in reducing osmotic stress [174], however others report an insignificant contribution of MAAs as compared to other osmolytes [145]. MAAs can also act as osmoprotectants under freezing conditions [143,175]. Due to their high nitrogen content, a role as nitrogen intracellular reservoirs [143,176,177]. In marine reefs, under nitrogen-limiting conditions starved corals have beed observed to preferentially accumulate MAAs in higher rates than protein and chlorophyll a (Chl a) [178]. Lastly, particularly in species establishing symbiosis with *Symbiodinium* zooxanthellae, MAAs together with other free amino acids have been proposed to facilitate the exchange of photosynthates between symbiont and host, and hence act as “host factors” [179].

##### Environmental Distribution of MAAs and Their Occurrence in Organisms

MAAs have been found from tropical (e.g., Reference [165]) to Antarctic waters (e.g., Reference [84]) and in a variety of organisms [180], spanning from cyanobacteria (e.g., Reference [181]), microalgae (e.g., Reference [182]), fungi (e.g., Reference [183]), as well as macroalgae (e.g., Reference [184]) and animals—invertebrates and vertebrates (e.g., References [185,186]). Among animals, protozoans, poriferans, cnidarians, platyhelminthes, nemerteans, polychaetes, molluscs, bryozoans, rotifers, arthropods, echinoderms, tunicates, and fish have also been reported to protect themselves from UV-R by MAAs [180]. To date, few reports have described the production of MAAs in bacteria [187,188,189]. In most organisms, there is an expected positive correlation between MAA concentration and solar UV-R: MAAs contents vary seasonally, peaking during the summer months [190], they diminish with depth [165,167,168,191,192], and under shaded conditions [157,193], as well as if UV-R irradiance is filtered out manipulatively [149,166]. Interestingly, some free-living dinoflagellates synthesize and release MAAs into the water, which was interpreted to contribute to the attenuation of UV-R during algal bloom events [194]. Organisms may further compensate the levels of dietary MAAs with behavioural traits to reduce UV damage. For example, the sessile anemone *Actinia tenebrosa* displayed larger seasonal fluctuations of MAAs than the mobile intertidal gastropod *Diloma aethiops* from New Zealand [195].

About 95% of tropical, 80% temperate and 82% of polar species studied have detectable amounts of MAAs [184,185,196]. Palythine (**7**), shinorine (**10**), mycosporine-glycine (**2**), porphyra-334 (**9**), asterina-330 (**1**), palythinol (**8**), palythene (**5**), and mycosporine-2-glycine (**3**) are by order the most common MAAs in marine biota, and are found in all latitudes. Other MAAs, instead, have more restricted distributions, but this might be biased, due to limiting representative data. Characteristically, mycosporine-glycine-valine (**11**) seems to be more associated to Antarctic ecosystems [185]. By phylum, cnidarians seem to possess the highest diversity, in part because there are more publications on this phylum [45]. In particular, scleractinian corals have attracted attention for their study, as they are the major bioconstructors of the impressive tropical reefs. MAAs are common in microalgal-invertebrate symbioses on coral reefs and other habitats [131]. At least 11 different MAAs have been reported in corals particularly in their mucous, being palythine (**7**) and mycosporine-glycine (**2**) the most abundant [118,169,170,197]. There are species reflecting dietary MAAs with presence exclusively in the coral host [126,161], while others require provision of these sunscreen products from their symbiotic zooxanthellae cells (*Symbiodinium*) [162,198], and/or prokaryotic partners (e.g., bacteria in the genus *Vibrio* sp.; [159]). The host organism may also play an important role in protecting symbionts from UV-R damage, for instance some ascidians accumulate MAAs in the tunic, which prevents photoinhibition of *Prochloron* symbionts [199]. In macroalgae (from tropics to the poles) shinorine (**10**) and porphyra-334 (**9**) are by far the most common MAAs. Red algae are the largest producers (Rhodophyceae—but with notable variability among species), followed by Phaeophyceae, and last a few green algae—Chlorophyte and Charophyte [84,180,200]. Banaszak et al. [170] however, reported mycosporine-glycine (**2**) as the most abundant MAAs in temperate macrophytes, especially in Chlorophyte and Phaeophyte.

The dietary uptake of MAAs can be selective [160,201]. For example, the sea urchin *Strongylocentrotus droebachiensis* principally accumulated shinorine (**10**) in the ovaries, but not palythine (**7**), asterina-330 (**1**) and usujirene (**11**) that were available in high concentration in their diet [201]. Moreover, subsequent biochemical or possibly bacterial (endosymbionts) conversion of acquired MAAs can sometimes increase the variability of these compounds in certain organisms [202]. This is likely the reason why some host species harbor more MAAs than their symbionts or prey [135,149,161]. On the contrary, when an organism contains fewer MAAs than the symbiont or its prey it is possible that some MAAs were not eventually incorporated or were degraded [140]. Photoprotectants can also be transmited vertically to the offspring, as a mechanism to favour larval survival [158]. For instance, in several species of soft corals MAAs contents exhibited a peak in female colonies prior to spawning in comparison to male counterparts (e.g., up to a 67% and 56% difference for *Lobophytum compactum* and *Sinularia flexibilis*, respectively) [190,203]. When embryos of some echinoderms fed on algal diets with richer MAAs contents, these obtained photoprotection against abnormalities induced by UV-B in the first stages of life [191,201,204]. Shinorine (**10**) indeed, was prevalent in the ovaries and eggs of sea urchins from polar to tropical biomes [170,185,191,201]. Other than to eggs and reproductive tissues, marine animals have been described to allocate MAAs to specific tissues—e.g., holothuroids in the epidermis [131,157], tridacnid clams in siphonal mantle and kidney [205], didemnid ascidians in tunic bladder cells [206], corals in mucous exhudates [207], and teleost fish in ocular tissues [160,186].

##### MAAs in Antarctic Marine Organisms

The deleterious effects of UV-R are presumed to be particularly acute in the Southern Ocean, and even more in the superficial layers of the water column [63]. Consequently, the planktonic fraction in this region is likely to rely much on MAAs and other photoprotective agents, and there are several studies corroborating this assumption (e.g., References [116,208,209,210,211,212,213,214]. Antarctic phytoplankton assemblages are rich in MAAs, especially when dominated by prymnesiophytes in the genus *Phaeocystis* [116,211], as well as by chain-forming *Thalassiosira* diatoms (e.g., *Thalassiosira gravida*; [209]). These assemblages exhibit though very different MAA compositions—e.g., *Phaeocystis antarctica* blooms have more complex profiles (mycosporine-glycine (**2**), shinorine (**10**), porphyra-334 (**9**), palythine (**7**), palythinol (**8**) and palythenic acid (**6**)), whereas those formed by *Thalassiosira* diatoms are dominated by shinorine (**10**) and porphyra-334 (**9**), and at times include mycosporine-glycine (**2**) [154,213,215]). Hernando et al. [154] found that UV-B induced the expression and accumulation of MAA in a *Thalassiosira* sp. diatom. Instead, more latitudinal cosmopolitan blooms, such as those formed by the prymnesiophyte coccolithophorid *Emiliania huxleyi* display restricted UV-R tolerance, linked with low concentrations of only shinorine (**10**) [153]. Contrasting to bloom-forming algae, MAAs seem to play a minor role as photoprotectants in sea ice algae, where they are found in low amounts. It has been suggested that the ice algal structure may provide a self-shading effect, thus confering protection on the community as a whole [216]. The Antarctic krill *Euphausia superba* recorded a variety of at least nine MAAs [84], likely from dietary phytolankton uptake [217]. However, it was particularly rich in rare isomeric forms of palythenic acid, suggesting *Z/E* isomerization during assimilation [218]. A characteristic case study among Antarctic plancto-pelagic organisms is that of the pteropod predator *Clione antarctica*, and its exclusive prey, the herbivorous pteropod *Limacina helicina*, both containing the same five MAAs, mycosporine-glycine (**2**), shinorine (**10**), porphyra-334 (**9**), palythenic acid (**6**), and palythine (**7**). Strickingly, the phytoplankton assemblage, on which *Limacina helicina* feeds on has only shinorine (**10**) and porphyra-334 (**9**) [135], suggesting that there is likely the possibility of subsequent biochemical or bacterial interconversions of shinorine (**10**) and porphyra-334 (**9**) [202], or perhaps other minor food sources.

Most of the knowledge about the use of UV protectants by subtidal benthic Antarctic marine organisms comes from the surveys of Karentz and co-workers [84,219] near Palmer Station and from McMurdo Sound areas. Regarding seaweeds, around 25 species have been studied for MAAs contents [84,219,220,221,222]. While shinorine (**10**) and porphyra-334 (**9**) are the most common in macroalgae from tropical to polar waters [84,180,184,223], Antarctic red algae (i.e., *Palmaria decipiens*, *Iridaea cordata*, *Curdiea racovitzae, Kallymenia antarctica*) showed more complex profiles, comprising shinorine (**10**) and porphyra-334 (**9**), palythine (**7**), asterina-330 (**1**), palythinol (**8**), palythene (**5**), usujirene (**11**) and the unusual M335/360 [84,180,219,220,221,222]. Such higher MAAs diversity may be consequence of interconversions among primary and secondary MAAs [202].

Karentz and co-workers [84,219] found in general low levels and abundances of MAAs in marine organisms inhabiting subtidal (>20 m depth) Antarctic seafloors from Palmer Station and McMurdo Sound. The reason for this trend was suggested to be trophic related, as the majority of those benthic species were not herbivorous. To the best of our knowledge, the species analyzed for MAAs contents so far include: 18 Porifera, 2 Cnidaria, 2 Platyhelminthes, 3 Nemertea, 15 Mollusca, 6 Annelida, 14 Arthropoda, 2 Bryozoa, 7 Echinodermata and 5 Chordata (of which three are ascidians and two are fish). Eleven MAAs (mycosporine-glycine, mycosporine-2-glycine, shinorine, porphyra-334, palythine, mycosporine-glycine-valine, asterina-330, palythene, palythinol, palythenic acid and usujirene (**1**–**11**)) were detected among these species, but four—mycosporine-glycine (**2**), shinorine (**10**), porphyra-334 (**9**) and palythine (**7**)—where present in >50% of the cases [84,219]. Similar as in some echinoderms from non-Antarctic latitudes, the ripe ovaries and testes of the sea urchin *Sterechinus neumayeri* and the ripe ovaries and brooded young of the sea cucumber *Cucumaria ferrari* were enriched in MAAs [219]. In a subsequent study monitoring temporal changes of MAAs in tissues and depth, *Sterechinus neumayeri* reported the highest concentrations in the ovaries, varing according to the spawning cycle and with depth [191].

Table A1 reports the list of MAAs found in Antarctic marine organisms available in the literature up to now.

#### 3.2.2. Scytonemin

**Figure 3:**
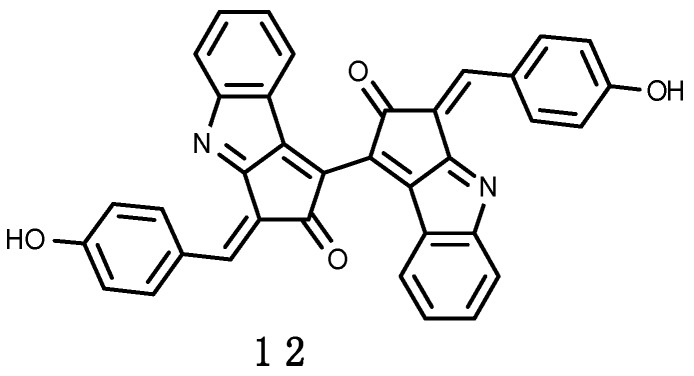
(**12**) Scytonemin.

Some terrestrial cyanobacteria living in habitats exposed to full sunlight produce scytonemin (**12**) [224], shown in Figure 3. It is a yellow-brown lipid soluble sheath pigment that absorbs maximally in the UV-A and UV-C regions, but with some absorbance in the UV-B region [225]. Scytonemin is composed of a dimeric structure of indolic and phenolic subunits having a molecular mass of 544–546 Da, with an in vivo absorption maximum at 370 nm. Both, temperature increase and oxidative stress combined with UV-A have a synergistic effect on the synthesis of scytonemin (**12**) [226]. In Antarctica the primary genera rich in sheath pigments are *Gloeocapsa* and *Calothrix*, which form black or brown crusts over rocky streambeds, and *Nostoc*, which forms black, mucilaginous films and mats up to several centimeters thick along stream banks and in slow moving waters [108]. Scytonemin (**12**) has been identified in *Nostoc commune* and *N. microscopicum* isolated from fresh water pond fringe in Mc-Murdo Ice Shelf, Antarctica [224]. Cyanobacteria may exist in Antarctic sea ice, but contributing insignificantly to the marine ecosystems [227]. They are instead important components of Antarctic terrestrial and freshwater microflora, colonizing rocks, and found in lakes, ponds, meltwater holes and streams [224].

#### 3.2.3. Erebusinone

**Figure 4:**
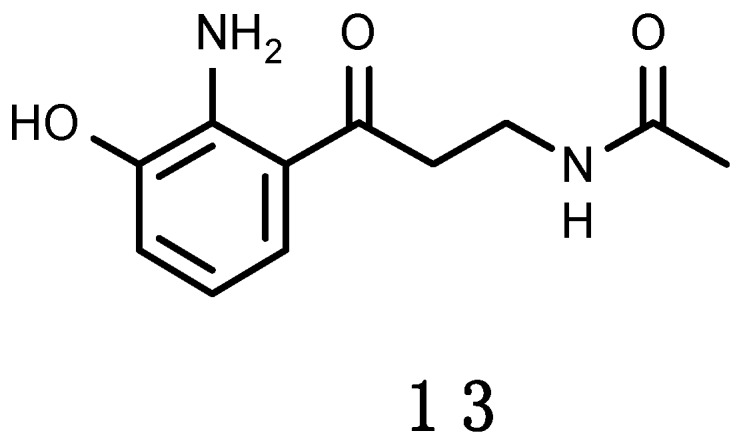
(**13**) Erebusinone.

A yellow pigment, erebusinone (**13**), shown in Figure 4, has been found in the Antarctic sponge *Isodictya erinacea* [228]. It shares the same aromatic substitution pattern of 3-hydroxykynurenine. 3-Hydroxykynurenine is a water soluble, low molecular weight, tryptophan derivative that occurs in the lens pigments of several species of marine and freshwater fish [229,230]. It absorbs in the UV-A region with a peak absorbance of 370 nm. Such photoprotective properties increase visual acuity by reducing glare, scatter and chromatic aberration while maximizing contrast, this way aiding prey detection or functioning as a stabilizing lens protein [230]. Erebusinone shares similar absorbance properties as 3-hydroxykynurenine, and biogenesis from the tryptophan catabolic pathway. Erebusinone (**13**) was evaluated for bioactivity against the sympatric predator amphipod *Orchomene plebs*, causing reduced molting and increased mortality at ecologically relevant concentrations. This seems to be the first example of molt inhibition as a mechanism of chemical defence in the marine environment [228].

Table A2 shows all known UV-absorbing compounds found in the Antarctic marine environment that are not MAAs.

#### 3.2.4. Pigments

**Figure 5:**
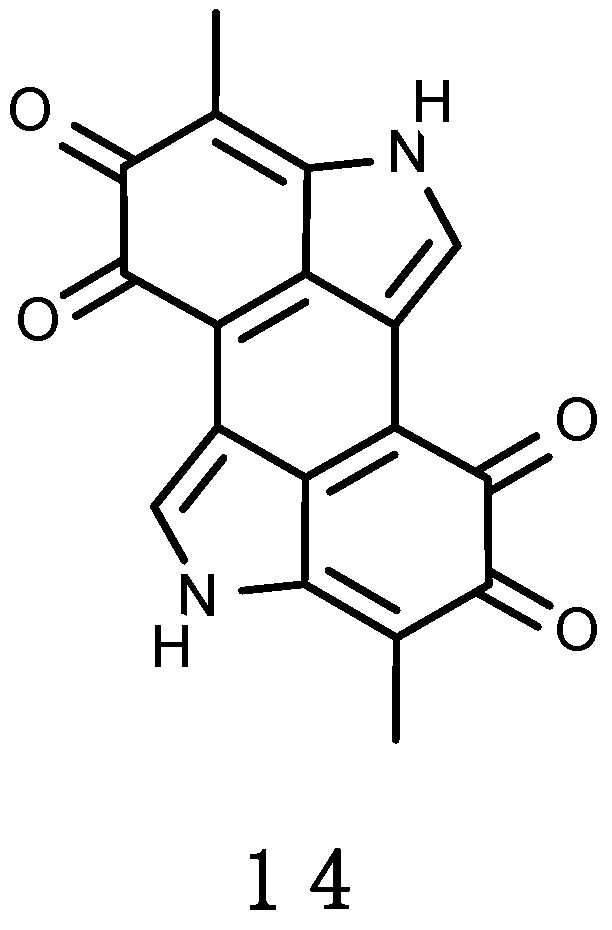
(**14**) Melanin.

Melanin (from the Greek *melas*, “black, dark”) (**14**), shown in Figure 5, is produced by the oxidation and polymerization of tyrosine. Melanin is a UV-absorbing compound and belongs to a group of pigments responsible for dark, tan, and even yellowish or reddish pigmentations, due to the aerobic oxidation of phenols. It is a polymer of either or both of two monomer molecules: Indolequinone and dihydroxyindole carboxylic acid [231].

Because melanin is an aggregate of smaller component molecules, by changing the proportion and bonding pattern of the component molecules, a wide number of different types of melanins can be produced [231]. It is an effective light absorbent at all UV-R and PAR wavelengths able to dissipate over 99.9% of absorbed UV-R [232], and thus is a wide-ranging sunscreen in non-photosynthetic organisms (e.g., Reference [233]). In the skin of fish (e.g., genus *Xiphophorus*), for instance, melanin could lower the rate of pyrimidine dimers formation caused by exposure to various UV-R [234]. Melanin can have diverse functions in various organisms. It has been described to act as a free radical scavenger and energy transducer, majorly in microbial fungi and bacteria [235,236,237]. Some arthropod species have deposits of melanin in layers that yield an iridescent color by alternating refractive index effect—Bragg reflector [238]. In invertebrates, an immune response to invading pathogens called “melanisation” has been described, by which microbes are encapsulated within melanin. The consequent generation of free radical byproducts is thought to aid in their elimination [239]. In cephalopods instead, melanin takes part of the ink as distracting-scape defence against predators [240].

*Alteromonas stellipolaris*, a bacterium from Antarctic seas, produces a brown-black pigment characterized as melanin [241]. Melanin has been also identified in *Lysobacter oligotrophicus*, a Gram-negative bacterium isolated from an Antarctic freshwater lake [242], and in black fungi found in the Antarctic terrestrial biotopes [243,244].

Examples of melanin found in the Antarctic marine environment are reported in Table A2.

Some other UV-absorbing pigments with no specified name were detected in the Antarctic algae *Palmaria decipiens* and *Enteromorpha bulbosa* [245]. Many pigments that confer bright colourations to Antarctic sessile organisms (e.g., sponges), such as the mentioned erebusinone (**13**) from *Isodictya erinacea*, but also variolins from *Kirkpatrickia variolosa*, discorhabdins in *Latrunculia apicalis*, suberitenones from *Suberites* sp., and the yellow isoquinoline pigment from *Dendrilla membranosa* possess intriguingly striking bioactivities. It has been proposed that such colourful molecules could be the result of relict pigments originally retained for aposematism or UV screening, and then conserved because of their beneficial defensive properties, such as feeding deterrents or antifouling (revised in Reference [231]).

#### 3.2.5. Other Secondary Metabolites

##### Phlorotannins

**Figure 6:**
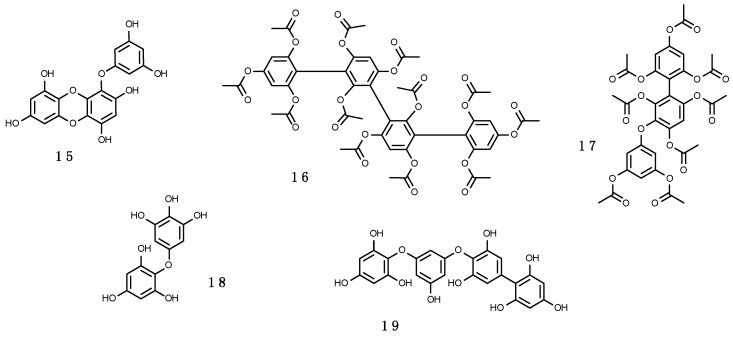
Examples of marine phlorotannins: (**15**) Eckol; (**16**) fucol; (**17**) fucophlorethol; (**18**) fuhalol; (**19**) phlorethol.

Phlorotannins are polymers of phloroglucinol (1,3,5-trihydroxybenzene), a type of tannins analogous to the shikimate-derived polyphenolics, including more than 150 compounds (e.g., (**15**–**19**), shown in Figure 6). Their molecular weight ranges from 10 to 650 kDa. They exhibit strong absorption from 280 to 320 nm [246,247] and their production is induced by exposure to UV-B, in some cases probably after external wounding or herbivory [248]. Their presence is particularly abundant in cell walls of brown algae (~10–20% dry weight; [246]) and can be found in low amounts in red algae. This may explain why MAAs are virtually absent in brown algae in comparison to red and green algae [84,170,220,249]. Phlorotannins protect algal cells against UV-R damage, but they can also be exudated in the surrounding water [246,250]. Moreover, phlorotannins may have several other roles (reviewed in Reference [251] including antioxidants as efficient ROS scavengers [252], antiherbivory defences (e.g., Reference [247]), structural function as part of brown algal cell walls and implicated in cytokinesis [249], reproduction agents in fertilization processes as spermatozoan inhibitors [253], wound healing factors [254], algicidal effect against some dinoflagellates [255], and heavy metals sequesters [246,256]. In fact, a combination of both UV-screening phlorotannins and major antioxidant enzymes may be used to respond to unfavourable light conditions [257]. As an example, it has been demonstrated that the influence of UV-R on biological processes is dependent on the exposure time: Short-term responses are mediated by down-regulation of the photochemical machinery and the increase in the synthesis of antioxidant enzymes, while long-term responses are mediated primarily by an increase in the induction of soluble phlorotannins [257].

Antarctic seaweeds—in particular endemic brown algae—reveal a tremendous bathymetric range (e.g., Desmarestiales, such as *Himantothallus grandifolius*, *Desmarestia anceps* and *Desmarestia menziesii*, and some fucoid species, such as *Ascoseira mirabilis* and *Cystosphaera jacquinotii* extend from 2 to 40 m), indicating a remarkable photobiological adaptation [258,259]. Furthermore, brown macroalgae are able to allocate large amounts of phlorotannins in selected parts of their thalli [247,248]. This strategical storage in algal fronds can change over short periods of time, with highest contents during low tides and higher UV stress in summer [260,261,262]. In fact, the synthesis and accumulation of phlorotannins and their antioxidant capacity were found to follow a diurnal course in several brown algae [261].

Several studies on Phaeophyceae, and also other common rhodophytes (e.g., *Iridaea* *cordata, Trematocarpus antarcticus* and *Palmaria* *decipiens*) reveal that such acclimatory flexibility to depth and seasonal patterns for light demands and UV tolerance appears to be strongly related to the high phlorotannin contents and antioxidant potential [262,263,264]. These functional traits provide benefits not only at an individual scale, but also explain both the stability and resilience capacity of the whole benthic community that depends on these organisms [264]. In relation to this, Iken et al. [265,266] found that nine abundant Antarctic brown algae contained higher levels of phlorotannins than most tropical and North Pacific species, but were comparable to levels in Australasian species. Rautenberger et al. [262] suggested that UV tolerance in macroalgae, which are sensitive to UV-R, is modulated by temperature, producing species-specific effects. But not all types of phlorotannins behave in the same way. Soluble phlorotannins exhibit a more dynamic short- and mid-term responses towards UV exposures, as observed in the kelps *Lessonia nigrescens,* *Lessonia spicata* and *Macrocystis pyrifera* and the fucoid *Durvillaea antarctica* [267,268,269,270]; whereas the insoluble content has a more stable pattern, displaying little differences upon variations of irradiance, depth or season [267,268,269]. Indeed, algae tend to anatomically allocate these two phlorotannins fractions accordingly, e.g., soluble phlorotannins tend to be more concentrated in reproductive and active structures than in vegetative tissues, where the insoluble phlorotannins are predominant [268,269].

Regarding their precise chemical structure, in contrast to hydrolysable and condensed tannins, which are easily analysed with separation methods, such as HPLC and capillary electrophoresis, phlorotannins are still commonly analysed as the total amount of the whole compound group by colorimetric methods using phloroglucinol as a standard agent [271]. In fact, current knowledge of their ecology is based almost entirely on the total contents of phlorotannins, since it usually is a very complex mixture, challenging structural elucidation of the individual compunds [272]. Examples of the diversity of phlorotannins in brown algae include fucols (with phenyl linkages) (**16**), phlorethols (**19**) and fuhalols (**18**), (with ether linkages), eckols (with dibenzodioxin linkages) (**15**), and fucophlorethols (with ether and phenyl linkages) (**17**). As far as we know, the reported Antarctic phlorotannins have not been identified to individual level so far.

##### Flavonoids

Flavonoids (from the Latin *flavus* = yellow) are a type of secondary metabolites found in plants and fungi. They have an absorption spectrum from 280 to 340 nm, providing UV screening, while allowing transmission of PAR for photosynthesis. Flavonoids are synthesized stimulated by UV-R through the phenylpropanoid pathway. These phenolic compounds fulfill many other roles, including resistance to predators and pathogens, pollinator enhancers, and seed dispersal agents [273]. Flavonoids are not reported in Antarctic systems, due to the lack of marine phanerogams.

##### Tridentatols

Tridentatols A to D are unique phenolic metabolites with an uncommon sulfur-containing functional group isolated from the hydroid *Tridentata marginata*, which lives commonly associated with the pelagic *Sargassum* community around the Caribbean. They display a strong absorption in the UV-A and UV-B regions ranging 313–342 nm, and have been hypothesized to function in photoprotection, as well as serving as deterrent agents from predators, thereby performing a dual role [274]. Similar compounds have not been reported in Antarctic organisms so far.

#### 3.2.6. Fluorescent Proteins

Green fluorescent-like proteins, widely distributed amongst symbiotic cnidarians, fluoresce in the presence of UV-R or PAR [275]. Highly resistant to extreme pH and temperature, they were originally isolated and described from the hydromedusae *Aequorea victoria* [276,277]. Moreover, their SOD-like activity can quench superoxide radicals [278]. To our knowledge, green fluorescent-like proteins have not been identified yet in Antarctic marine organisms.

### 3.3. Quenching Mechanisms

Once UV-R reaches the inside the cell, it interacts with O_2_ and other organic compounds to produce harmful ROS, such as superoxide (O_2_**^−^**•), hydroxyl radical (•OH), hydroperoxyl radical (HO_2_^−^) or hydrogen peroxide (H_2_O_2_) provoking oxidative stress. ROS can damage important biomolecules, such as DNA, proteins and lipids [279]. Polyunsaturated fatty acids (PUFAs) are one of the primary targets of ROS by removing a proton from conjugated double bond systems, forming a peroxyl radical that then activates lipid peroxidation chain reactions [280], causing tissue damage [281] and alterations of the integrity of cell membranes [282]. This is of particular relevance for marine organisms living in Antarctica, since they have a high content of PUFAs to improve membrane fluidity at low temperature [283]. Differences in PUFAs and in their relations to other fatty acids were also described between Antarctic and Mediterranean gastropod molluscs [284]. To counteract the oxidative stress by ROS, marine organisms have evolved antioxidant systems based on both non-enzymatic and enzymatic antioxidants. Examples of Antarctic marine antioxidants are reported in Table A3.

#### 3.3.1. Non-Enzymatic Antioxidants

##### Carotenoids

Carotenoids are structurally and functionally very diverse natural pigments and important components of the photosynthetic apparatus, playing a dual role by: (i) Enhancing cellular photosynthetic production, and (ii) providing photooxidative protection. They are derived from five carbon isoprene units that are polymerized enzymatically to form regular highly conjugated 40-carbon structures with up to 15 conjugated double bonds [285]. They are grouped in two main classes: carotenes, which are hydrocarbons that may go through cyclization to form β-ionone ring end groups, which additionally may be substituted by oxo, hydroxy or epoxy groups at dissimilar positions to form different xanthophylls, oxygenated derivatives of the former carotenes, which constitute the other classe of carotenoids [286].

**Figure 7:**
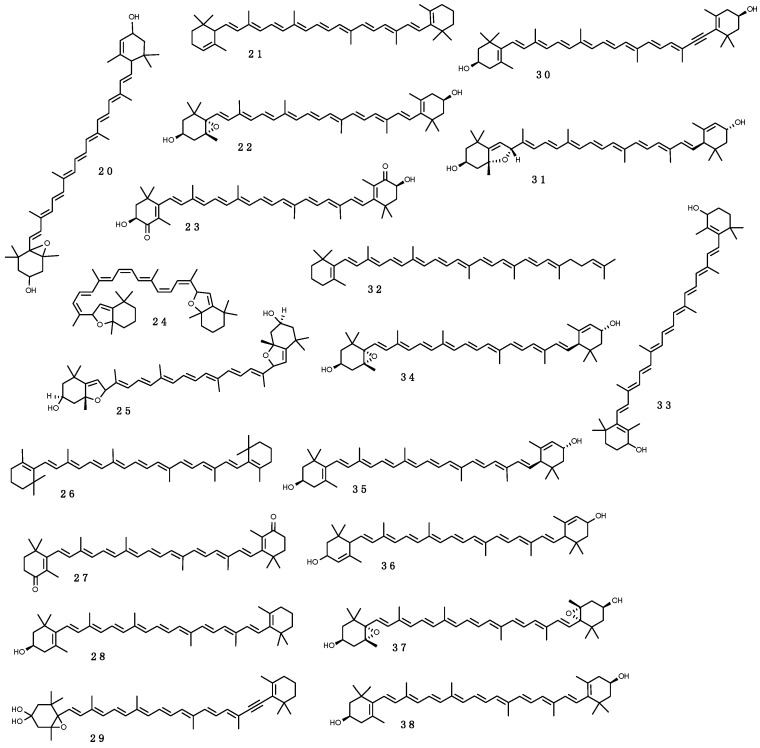
Carotenoids: (**20**) 4-hydroxy-α-carotene; (**21**) α-carotene; (**22**) antheraxanthin; (**23**) astaxanthin; (**24**) aurochrome; (**25**) auroxanthin; (**26**) β-carotene; (**27**) canthaxanthin; (**28**) cryptoxanthin; (**29**) diadinoxanthin; (**30**) diatoxanthin; (**31**) flavoxanthin; (**32**) γ-carotene; (**33**) isozeaxanthin; (**34**) lutein-5,6-epoxide; (**35**) lutein; (**36**) tunaxanthin; (**37**) violaxanthin; (**38**) zeaxanthin.

One of the most characteristic features of carotenoids is their strong coloration, which is a consequence of light absorption, due to the presence of an extensive system of conjugated double bonds, which is crucial for the proper functioning in light absorption in photosynthetic organisms and photoprotection in all living organisms [287]. Nearly all carotenoids absorb light in the 400–500 nm range, generating their typical UV and visible spectra, with three absorption maxima [286]. The best studied carotenoids include α-, β- and γ-carotene (**21**, **26**, **32**), lutein (**35**), zeaxanthin (**38**), violaxanthin (**37**), diadinoxanthin (**29**), diatoxanthin (**30**), anteraxanthin (**22**), astaxantin (**23**), and flavoxanthin (**31**), shown in Figure 7. Their quenching properties allow dissipate excess energy from UV-B, which would otherwise generate toxic single O_2_, thus protecting the photosynthetic machinery from irreversible inhibition. Energy dissipation in light-harvesting antenna systems occurs via direct energy transfer from the Chl an excited state to the carotenoid S1 (lowest excited) state. The safe dissipation of excess energy as heat, also observed as a reduction in fluorescence, is a process known as non-photochemical quenching [288].

Under excess light condition, the formation of a pH gradient across the thylakoid membrane activates the xanthophyll cycle, consisting in the reversible de-epoxidation of violaxanthin (**37**) to zeaxanthin (**38**) and anteraxanthin (**22**) [289,290]. The amount of excitation energy dissipated by this process depends on the pool size and on the de-epoxiation state: More epoxides (violaxanthin (**37**)) means less energy dissipation and less photoprotection. The epoxidation state (epoxidated/(epoxidated + de-epoxidated pigments)) can, therefore, be considered an indicator of xanthophyll cycle activity under excess irradiance conditions [291].

Increases in xanthophyll/Chl a ratio is commonly observed in microalgae and macroalgae and higher plants subjected to high irradiance, including UV-R [291].

Carotenoids with photoprotective activity have been described in the Antarctic red algae *Leptosomia simplex* [245]. Among Antarctic microalgae, *Polarella glacialis* was shown to possess a very high xanthophyll/Chl a ratio, together with a high content of UV-absorbing compounds, at the highest PAR acclimation levels, appearing thus well equipped to cope with high irradiance. In addition, low intracellular concentrations of the lipid peroxidation by-product malondialdehyde were observed in this species, possibly indicating that antioxidant mechanisms are able to prevent rapid accumulation of harmful oxy-radicals that could otherwise oxidise cellular membranes [291]. It is worth to mention that UV-B vulnerability is known to be species specific, although may also be affected by a range of environmental growth conditions, including the light history of the cells. For example, under fixed light, MAA synthesis seems the most effective photoprotective mechanism activated by a microalga *Eutreptiella* sp. from the Southern Ocean, which under variable light conditions was instead successfully protected by the synthesis of photoprotective pigments of the xanthophyll cycle [292].

A study conducted on the Antarctic microalgae *Chaetoceros dichaeta*, *Pyramimonas gelidicola*, *Phaeocystis antarctica* and *Polarella glacialis,* confirmed species-specific sensitivity to UV-B and CPDs formation, also showing that acclimation to high PAR induced an increased sensitivity of the species *Pyramimonas gelidicola* to UV-B with a consequent increase of DNA damage [291].

In high irradiance acclimated cells of the marine diatoms *Thalassiosira weissflogii* and *Thalassiosira antarctica*, the diadino-diatoxanthin (**29**–**33**) pool was increased compared with cells grown under low irradiance [293]. These authors also suggested that light harvesting pigment ratio is a sensitive indicator of excessive irradiance sensitivity, and small species-specific differences in pigment composition affect photo-induced viability loss.

Carotenoids with an absorption peak at 384 nm were found in seven-year old sample of cyanobateria of the genera *Nostoc*, from coastal lowland adjacent to the Ross Ice Shelf [294]. Survival to UV-stress of *Nostoc* spp. and other cyanobacterial species common in habitat fully exposed to maximum UV-B during the ozone minimum of early spring, may be due partly to their carotenoid content. Antarctic benthic mats of cyanobacteria contain high concentrations of carotenes and xanthophylls [295,296] with the highest concentrations in the upper surface strata that confers the bright orange or pink coloration of many of these Antarctic communities [108]. Some pigmented bacteria of Antarctic soil samples owe their colors to the presence of carotenoids [297]. A similar phenomenon is described for Antarctic marine bacteria *Antarcticimonas flava* [298] and *Muricauda antarctica* [299], which are marine members of the Flavobacteriaceae isolated from Antarctic seawater, and from heterotrophic bacteria isolated in water samples from lakes and supraglacial fluvial system [300]. Other examples include the Antarctic cyanobacteria *Anabaena*, *Nostoc* and *Phormidium,* which contain higher carotenoid content than their corresponding tropical strains [301]. Also, the Antarctic algae *Delesseria lancifolia* showed a more complex xanthophyll pattern respect to species from other areas, with violaxanthin (**37**), antheraxanthin (**22**), and zeaxanthin (**38**) as major compounds, and without evidence of chlorophylls other than Chl *a* [302]. All these xanthophylls are derivatives of β-carotene (**26**), indicating that the alga is unable to perform the α-cyclization of lycopene in the biosynthesis pathway [302].

Aquatic animals contain significant amounts of carotenoids derived from dietary source, primary from algae and as secondary source from other animals, which accumulate the pigments from phytoplankton. More than 100 carotenoids have been isolated from sponges, cnidarians, molluscs, crustaceans, echinoderms, tunicates and fishes [303]. Antarctic krill (*Euphausia superba*), and other Antarctic zooplankton, accumulate in the head and shell significant amounts of carotenoids, especially astaxanthin (**23**), deriving from their algal food and use them as antioxidant or photoprotector [217]. Antarctic krill is considered as a new alternative, sustainable source of antioxidants, such as astaxanthin (**23**), vitamins A and E, and long chain n-3 PUFA [304,305].

The presence of various carotenoids was evaluated in certain species of Antarctic fish as in the *Rajidae (Raja georgiana), Muraenolepidae (Muraenolepis microps), Notothenidae (Dissostichus eleginoides, Notothenia gibberifrons, Notothenia rossi-marmorata, Trematomus hansoni)* and *Channichthyidae (Chaenocephalus aceratus, Champsocephalus gunnari, Pseudochaenichthys georgianus)*. Carotenoids identified were: β-carotene (**26**), α-cryptoxanthin (**28**), canthaxanthin (**27**), flavoxanthin (**31**), isozeaxanthin (**33**), zeaxanthin (**38**), tunaxanthin (**36**), lutein-5, 6-epoxide (**34**), aurochrome (**24**), aurochrome-like, auroxanthin (**25**), astaxanthin (**23**), astaxanthin ester and 4-hydroxy-α-carotene (**20**). The total carotenoid content of these fishes ranged from 0.066 to 0.122 µg/g fresh weight [306]. Finally, an astaxanthin-protein-calcium carbonate complex was purified from the aragonite skeleton of the coral Errina antarctica [307].

##### α-Tocopherol

**Figure 8:**
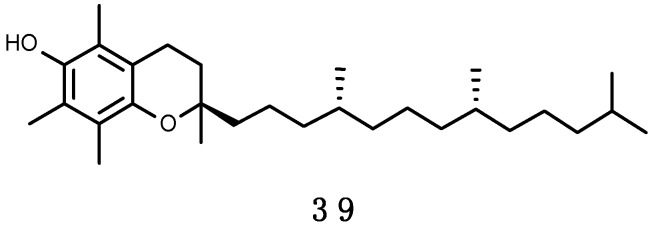
(**39**) α-tocopherol.

α-tocopherol (vitamin E) (**39**), as shown in Figure 8, is a non-enzymatic antioxidant. It refers to a group of lipid-soluble compounds that include both tocopherols and tocotrienols, produced exclusively by plants, algae, and some cyanobacteria [308]. The lipid soluble antioxidant α-tocopherol (**39**) is the most biologically active form of vitamin E, and it is located in the thylakoid membranes of photosynthetic organisms, where counteracts the effects of ROS by removing oxidized substrates or stopping the lipid peroxidation chains initiated by ROS [309]. Vitamin E acts as a peroxyl radical scavenger, disabling the production of damaging free radicals in tissues, by reacting with them to form a tocopheryl radical, which will then be reduced by a hydrogen donor (e.g., vitamin C) and thus return to its reduced state. Interestingly, vitamin E is not only a radical scavenger, but has a variety of other effects, which could also be an explanation for its photoprotective capacity. Initially, vitamin E absorbs UV light with a maximum at 295 nm [310], which is not often considered in the discussion of its photoprotective effect. Moreover, the inhibition of prostaglandin formation by vitamin E, resulting in the inhibition of inflammatory processes, might be associated with another photoprotective activity of this agent from the damaging effects of UV exposure in animals [311].

In the diatom *Thalassiosira* sp., isolated from natural phytoplankton assemblages from Potter Cove (Antarctic Peninsula) α-tocopherol showed a marked decrease during the exponential growth phase after exposure to solar UV-R, while the initial content of β-carotene did not show significant differences over time [312]. In addition, MAA production increased, suggesting that for this species photoprotection against UV-induced damage is characterized by short-term consumption of α-tocopherol and longer-term synthesis of MAAs. The UV-B damage/repair ratio during long-term exposure involves the combined action of several endogenous factors within the cell, with MAAs synthesis being the most effective factor related to photoprotection.

A higher content of α-tocopherol and β-carotene was also found in the Antarctic bivalve *Laternula elliptica* with respect to the temperate mud clam *Mya arenaria*. The higher vitamins content mirrored lipid radical content that was higher in *Laternula elliptica*. This study showed that lipid peroxidation extent in Antarctic bivalves can also depend on iron availability [313].

Biochemical adaptation of cellular membranes to function at low-temperature implies a corresponding need for enhanced lipid-phase antioxidant protection, and this is demonstrated by the increased need for dietary vitamin E by cold-water teleosts [314]. Antarctic fish, that have a high content of PUFAs in the plasma membrane to improve membrane fluidity at low temperature [283], are at an elevated risk of UV induced oxidative stress. To avoid lipid peroxidation, fishes and also invertebrates use vitamin E obtained from the diet as the most efficient lipid soluble antioxidant [315]. Vitamin E concentrations were five to six times higher in the Antarctic fish species, *Pagothenia borchgre*v*inki* and *Trematomus bernacchii* than in the plasma from two New Zealand temperate water fish species, blue cod (*Parapercis colias*) and banded wrasse (*Notolabrus fucicola*) [316].

**Figure 9:**
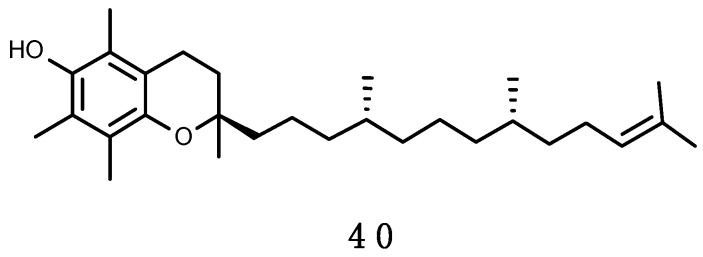
(**40**) α-tocomonoenol.

α-tocopherol was found to co-exist with a new derivative of vitamin E (α-tocomonoenol (**40**), shown in Figure 9) in Antarctic notothenioid fish *Chaenocephalus aceratus*, *Champsocephalus* *gunnari* and *Gobionotothen gibberifrons* and extracts of Antarctic krill *Euphausia superba*, as well as phytoplankton collected from the Antarctic Peninsula, ranging from 2.8 to 22.3% of the total vitamin E composition [317].

α-tocomonoenol bears an unusual methylene unsaturation at the isoprenoid-chain terminus, and it was firstly isolated from salmon eggs [318], being specific of marine organisms. Due to its specific occurrence, this antioxidant compound was named “marine-derived tocopherol” (MDT). MDT has a broader distribution in marine organisms with comparatively higher concentrations occurring in fish inhabiting cold-water environments, suggesting a specific metabolic function in low temperature adaptation [315]. MDT has greater reactivity with peroxyl radicals than α-tocopherol at low rates of radical flux and performed better than α-tocopherol also in preventing peroxidation of high-density, cholesterol-incorporated liposomes.

##### Ascorbic Acid

**Figure 10:**
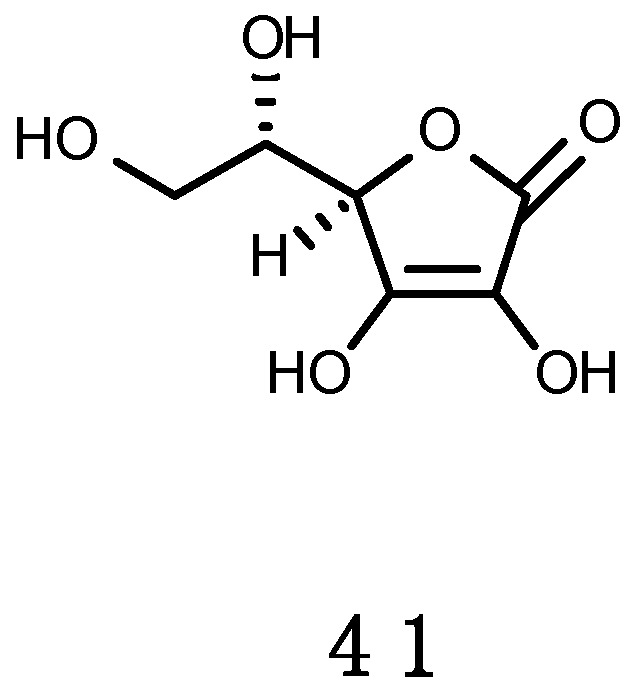
(**41**) Ascorbic acid.

Ascorbic acid (**41**), shown in Figure 10, is a water-soluble six-carbon compound related to glucose. Its biologically active form, vitamin C, functions as a potent reducing and antioxidant agent and as co-enzyme in several metabolic pathways [319]. It is synthesized by all species except for higher-order primates, guinea pigs, and some bat, fish, and bird species that consequently rely on a supply of vitamin C from their diet [320]. Apart from its antioxidant role, vitamin C also functions as cofactor for the enzymatic reduction of violaxanthin (**37**) to antheraxanthin (**22**) and zeaxanthin (**38**) involved in thermal dissipation of excess energy [321], and in the regeneration of vitamin E [316].

In Antarctic species, ascorbate content may significantly decrease after UV-B irradiation, as for the Antarctic *Chlorella* sp., cells in which a concomintant decrease of the α-tocopherol, β-carotene and total thiols contents was recorded, leading to the onset of oxidative stress state in its cells [322].

Vitamin C was found to be higher in the plasma of the benthic Antarctic fish *Trematomus bernacchii* than those found in the plasma of the cryopelagic *Pagothenia borchgre*v*inki* and of two New Zealand temperate water fish species, *Parapercis colias* and *Notolabrus fucicola* [316].

##### Glutathione (GSH)

**Figure 11:**
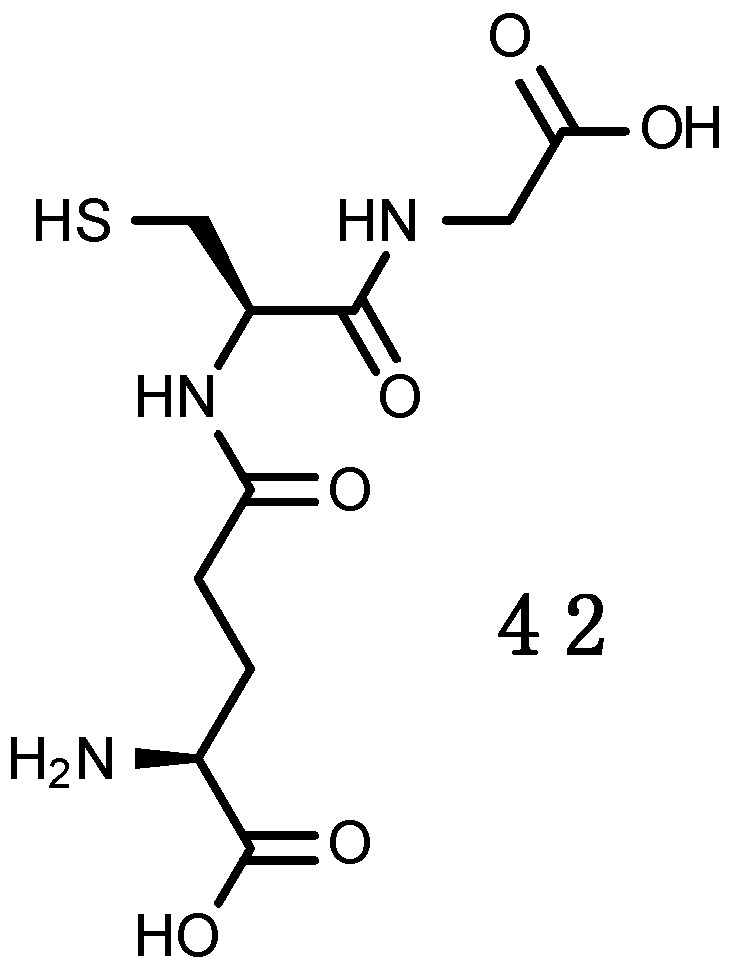
(**42**) Glutathione.

GSH (**42**), shown in Figure 11, is a tripeptide thiol involved in the maintenance of the cellular redox homeostasis [323], protecting thiol groups in various enzymes and with a relevant role in cellular protection by conjugation with toxic compounds or by quenching ROS. It is also involved in α-tocopherol (**39**) and ascorbate regeneration through the glutathione-ascorbate cycle [324] and it is in turn regenerated via the pentose phosphate pathway GSH [325]. Together with vitamin C is one of the main hydrophilic antioxidants, found in cytosolic, mitochondrial, chloroplastic, and nuclear aqueous compartments [326]. In non-cold-acclimated terrestrial plants, GSH protects from damage at low temperatures, but in cold-acclimated plants it is required in recovery from high temperature-induced damage suggesting a role for repair and redox homeostasis [327].

#### 3.3.2. Enzymatic Antioxidants

**Figure 12:**
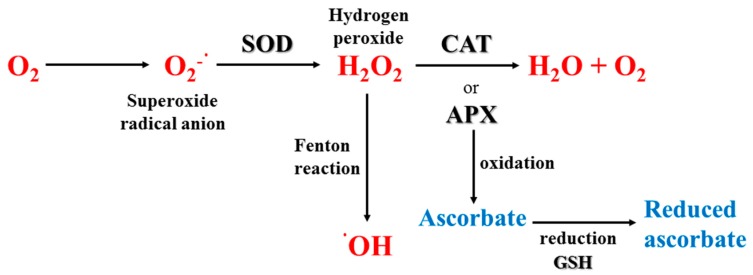
Antioxidant enzymes involved in the detoxification of reactive oxygen species (ROS). SOD, superoxide dismutase; CAT, catalase; APX, ascorbate peroxidase; GSH, glutathione.

The enzymatic antioxidants comprise SOD, CAT, glutathione peroxidase, glutathione-S-transferase and the enzymes involved in the ascorbate-glutathione cycle to detoxify ROS, such as ascorbate peroxidase, monodehydroascorbate reductase, dehydroascorbate reductase and glutathione reductase [328]. SOD scavenges superoxide radicals and converts them to H_2_O_2_, which is then converted to water and O_2_ via a combined CAT-peroxide system [329], (Figure 12). The ascorbate-glutathione cycle is the most important antioxidant cycle, especially in plants. The process starts with the production of the first ROS, the superoxide radical anion (O_2_**^−^**•), which is dismutated to H_2_O_2_ by SOD. The H_2_O_2_ is then reduced to H_2_O by ascorbate peroxidase (APX). The ascorbate oxidized by APX is reduced by the reduced form of GSH, which is yet again reduced by glutathione reductase [330], (Figure 12).

The SOD, peroxidase and CAT activities in the Antarctic sea ice microalgae *Chlamydomonas* sp. ICE-L were seen to be enhanced under UV-B radiation stress [105]. SOD seems to be an important ROS scavenging mechanism also in the Antarctic marine diatom *Chaetoceros brevis*, because the activity of SOD was rapidly up-regulated (4 h) in response to irradiance transitions [331]. In Antarctic clam *Laternula elliptica*, antioxidant capacities (CAT and GSH) are higher than those found in the temperate mud clam *Mya arenaria* whereas the level of lipofuscin, marker of oxidative damage, is lower in the polar than in the temperate clam [332]. To date, the majority of studies on antioxidant enzymes have been done on Antarctic fish [333,334,335,336,337], where additionally, the antioxidant enzyme system plays an important role in the adaptation to low temperature. As O_2_ solubility is inversely related to temperature, the Southern Ocean is O_2_ rich, with concentrations approximately 1.6-fold higher than in seawater at 20 °C [11]. From the biological standpoint, the advantage of a higher availability of O_2_ for oxidative metabolism is counterbalanced by the formation of high levels of ROS that are added to those produced by UV-R. Therefore, Antarctic marine organisms have an effective and intricate network of defence mechanisms that protects them against oxidative stress [338] and references herein. Transcript levels of proteins involved in antioxidant metabolism and multiple gene copies of ROS scavengers, as well as proteins that mediate iron processing—as iron promotes the production of free radicals—are higher in Antarctic fish than in warm bodied counterparts. Among the proteins involved in the response to oxidative stress, hemoproteins play a relevant role in Antarctic marine organisms [339].

### 3.4. Mechanisms of Repair from Photodamage

When neither physical nor chemical barriers are able to avoid or reduce UV-induced damage, a variety of repair mechanisms and resynthesis of sensitive targets are carried out to restore the cellular components after UV damage.

DNA is the primary lethal target and its damage is wavelength dependent: UV-A causes indirect damage to DNA by radicals (e.g., singlet O_2_, hydroxyl ions) in intra- or extracellular fluids [340], damaging also proteins and lipids. UV-B and UV-C cause both indirect and direct damage, because of the strong absorption of DNA at wavelengths below 315 nm. In Antarctic environments, the cool temperatures promote a decrease in the effectivity of repairing processes of damaged molecules, and adverse UV impact may be further exacerbated with episodes of ozone depletion [27]. Molecules involved in the mechanisms of repair—of DNA, protein, and others—developed in Antarctic marine organisms against UV-B damage are listed in Table A4.

#### 3.4.1. DNA Repair

The main target of solar UV-B radiation is DNA, whose peak of absorbance is at 260 nm maintaining a good absorption into all UV-B range [3]. When DNA absorbs UV light, structural changes occur in the molecule that, if uncorrected, can interfere with DNA synthesis and RNA transcription, with consequences for the translation of the genetic code. Besides loss of bases and single- or double-strand breakage, the most common UV-B-induced damage is represented by CPDs, pyrimidine 6-4 pyrimidone photoproducts (6-4PPs) and Dewar isomers [341]. CPDs are the most frequent lesions causing a block in DNA replication, impairing the normal function of replication of DNA, the transcription of genes and the synthesis of proteins [118,342,343]. The Antarctic ozone reduction may strongly increase the risk for CPD accumulation in the bulk of bacterio and phytoplankton, with consequences on the reduction of community growth and the loss of biomass from the water column [344]. In the Antarctic Ryder Bay, Buma et al. [344] demonstrated that, during mid-summer (January, beginning of February 1998), high levels of CPD were detected in the plankton with the highest found in the smallest size fraction, consisting of heterotrophic bacteria. Surface levels of CPD exceed 100 CPDs per million nucleotides in the bacterioplankton fraction. At the end of February and beginning of March, DNA damage was homogeneously distributed over the first 10 m, with levels between 20 and 30 CPDs per million nucleotides for the smallest size fraction. In the Antarctic nototheniod fish *Notothenia coriiceps* (family Nototheniidae; rockcods) and *Chaenocephalus aceratus* (family Channichthyidae; icefishes) and in the krill *Euphausia superba* high levels of CPD have been measured during periods of increased UV-B flux [103]. The capacity for DNA repair in Antarctic organisms has been shown to be highest in those species whose early life history stages occupy the water column during periods of ozone depletion (austral spring), and lowest in species whose eggs and larvae are abundant during winter [103]. In some Antarctic macroalgae—*Desmarestia* *menziesii*, *Ascoseira* *mirabilis*, *Desmarestia* *anceps*, *Iridaea* *cordata*, *Trematocarpus antarcticus*, *Palmaria* *decipiens* and *Himantothallus* *grandifolius*—, sensitive to UV-R at 2 °C, enhanced UV tolerance at 7 °C may be due to a more efficient damage repair of the photosynthetic apparatus rather than to an enhanced UV screening or radical scavenging [262].

Mechanisms of DNA repair represent an evolutionary ancient defence among organisms [345,346,347], indicating a very early requirement in the evolutionary history of life. In cyanobacteria, the unusually large number of DNA copies may give an additional protection against long-term UV-B damage, particularly in cold polar environments where the rates of DNA repair may be significantly decreased by the low temperature [108]. Common repair mechanisms include photoreactivation and excision repair (dark repair), which are the major pathways used to remove UV-induced DNA lesions, and others, such as recombinational repair and dimer bypass [3].

##### Photoreactivation

Photoreactivation can remove DNA lesions, such as CPDs or 6-4PPs, thanks to the activity of photolyase, flavin-dependent enzyme, that recognizes and binds to the DNA lesion and, using UV-A and blue light energy, splits the dimers and reverses the lesion [118]. Photolyases are ancient enzymes widely distributed in eukaryotes, eubacteria and archaea [348] but missing in placental mammals [349,350]. They contain a chromophore, such as 5,10-methenyltetrahydrofolate [351], or 8-hydroxy-5-deaza-riboflavin [352], able to absorb blue light and transfer the energy [353] to a non-covalently bound flavin adenine dinucleotide (FAD), which subsequently transfers an electron to the dimer, restoring the normal configuration of DNA [354]. Photolyases have been identified in the Antarctic marine bacteria *Pseudomonas*, *Janthinobacterium*, *Flavobacterium*, *Hymenobacter* and *Sphingomonas* [3,355], in different species of Antarctic diatoms [96], in the microalga *Chlamydomonas* sp. ICE-Lis [356], in the bipolar terrestrial moss *Sanionia uncinata* (Hedw.) Loeske [357], in the notothenioid fish, *Notothenia coriiceps* and *Chaenocephalus aceratus*, and in the krill *Euphausia* *superba* [103]. In the Antarctic sea urchin *Sterechinus neumayeri*, photoreactivation is the primary mechanism of removing CPDs, being able to repair all CPDs in less than 24 h [358]. In this organism, photolyase is constitutively expressed in all tissues and also induced in embryos in response to in situ exposure to UV-R, especially in shallower water depths or sea ice-free regions [359].

##### Dark Repairs

Another mechanism of DNA repair, common in mammalian cells and also present in prokaryotes and eukaryotes, is the nucleotide excision repair (NER). This is a light-independent mechanism, which involves a series of DNA replication enzymes that recognize DNA lesions and replace damaged DNA with new undamaged nucleotides. Endonuclease cuts the DNA strand at the lesion, DNA polymerase resynthesizes the correct sequence using the complementary undamaged strand of DNA, exonuclease cuts the damaged fragment, and ligase closes the new DNA strand. This kind of repair mechanism is present in bacterial isolates from Antarctic waters and different species of Antarctic diatoms [3]. *Xeroderma pigmentosum* protein C and RAD23 are crucial proteins of NER in metazoans, and are involved in damage recognition and the recruitment of repair factors [360,361]. Recently, the transcriptome of a sea anemone revealed full-length sunlight stress response genes codifying a chromoprotein and a photoprotein as gene families in cnidarians, while CPD photolyase and UV excision repair RAD23 were present as single copy genes [362].

In bacteria, but not in eukaryotic cells [363], NER together with postreplication recombinational repair and error-prone repair, belong to the dark repair mechanisms light-independent pathways that are triggered by the SOS response, well described in *Escherichia coli* [364]. Induction of the SOS response involves more than forty independent SOS genes, most of which encode proteins involved in protection, repair, replication, mutagenesis and metabolism of DNA [364]. However, there are two crucial proteins involved, LexA repressor and RecA, that can regulate transcription of cell cycle and DNA repair genes, and correct DNA damage [343]. When DNA damage is little, the genes of the SOS response are transcribed at basal level, due to the binding of the LexA repression protein to SOS promoters [365]. When large amount of DNA damage accumulates within cells inhibiting replication, the RecA protein activates the autocatalysis of the LexA protein, the SOS promoters are activated, and transcription of SOS genes occurs [366]. In Antarctic microbial populations, the timing of induction of genes involved in the SOS response is an important factor in the level of UV-tolerance SOS response [104]. The expression of recA gene and quantification of RecA protein have been used as an indicator of repair in Antarctic marine bacterioplankton communities. Levels of RecA protein vary during the day. It accumulates after sunset when it is still inactive, and it is consumed during the night as it operates [367].

#### 3.4.2. Other Mechanisms of Repair and Defence

In addition to repairing DNA, organisms can replace damaged proteins by de novo synthesis. Photosystem II (PSII) repair cycle and the de novo synthesis of the D1 protein are an important defence mechanism in UV-B-tolerance of Antarctic phytoplankton communities [368], as well as of the Antarctic cyanobacterium *Synechocystis salina* and the Antarctic green alga *Chlorella vulgaris* from moss and soil samples, respectively [369]. PSII is a protein-pigment complex involved in the electron transfer during photosynthesis that releases O_2_ catalyzing the transfer of electrons from water to plastoquinone, and D1 protein, located at its core has a rapid, light-dependent turnover [370]. Under all light intensities, the PSII repair mechanism is continuously active [371], but under UV-R stress, the D1 protein must be rapidly degraded and synthesized de novo via the PSII repair cycle to prevent the accumulation of damaged PSII and maintain the pool of active PSII [372,373]. Apostolova et al. [369] monitored the changes in the photochemistry of PSII of the Antarctic cyanobacterium *Synechocystis salina* and the Antarctic green alga *Chlorella vulgaris* and their mesophilic counterparts and demonstrated that the PSII activity of cyanobacteria was more vulnerable to UV-B radiation than that of green algae, whereas the mesophilic strain of *Synechocystis salina* was more susceptible to UV-B radiation than the Antarctic isolates.

Another mechanism used by the cells to respond to stressful conditions is to synthesize heat shock proteins (Hsps). The expression of Hsps in response to UV is assumed to be part of the endogenous UV response [374]. They are known to function primarily as molecular chaperones to assist protein folding/refolding of non-functional proteins [375], but are now recognized as sensors of cell stress in many systems [376] and as promoters of damaged proteins degradation, contrasting the toxic effects due to exposure to heat, UV-R, and other environmental stressors [377,378]. Hsps increase the resistance of cells to stress by influencing the DNA repair mechanisms or the induction of apoptosis [379]. Small Hsps have protein-protective activity and the capacity to stabilize lipid membranes [380]. UV-B radiation is able to increase the expression level of cytosolic Hsp70 gene in the Antarctic ice algae *Chlamydomonas* sp. ICE-L [381]. The Antarctic psychrophilic marine ciliate *Euplotes focardii*, a model organism well adapted to cold, is unable to respond to heat stress with the activation of Hsp70 genes, but under UV stress it is able to induce their expression [382].

When the cellular damage is too high to be repaired, apoptosis can occur to activate a programmed cell death, protecting the whole organism and sacrificing an individual cell [383,384]. Apoptosis in response to UV damage has been reported for several marine organisms [385], including various phytoplankton species [386], green algae (e.g., *Dunaliella tertiolecta*), and dinoflagellates (e.g., *Peridinium gatunense*) [387]. UV-induced oxidative stress is also known to play a role in apoptosis in sea urchin embryos activating genes, such as p53 and p21 [101].

## 4. Conclusions and Future Perspectives

In response to UV-R several photoprotective, quenching and repair mechanisms have evolved and persisted across many taxonomic groups as discussed in the above sections (and previous reviews, e.g., References [118,343]). Actually, the need of evasion from UV-R is one of the reasons for suggesting an origin of life in submarine vents, benthic crevices, and in deep environments [51]. The environment in future scenarios is pronosticated to continue a trend of global warming and ocean acidification, which will result in faster degradation of DOM, in particular CDOM. This will potentially enhance the penetration of UV-R, and in particular UV-B into the water column, emphasizing the need of marine ecosystems to retain photoprotective traits [388].

Antarctic species use the same mechanisms for protection and repair from UV as species thriving at lower latitudes [3]. However, the lack of data on species-specific responses to increased UV-B represents a limitation to the quantitative evaluation of the tolerance mechanisms with respect to other ecosystems. In this sense, the ecological impact of environmental harsh polar conditions and the ozone depletion in this region may favour towards the selection of more efficient biomolecular pathways for the production of photoprotectants. All in all, the photobiology of marine organisms from the Southern Ocean is still poorly explored and characterized by old specialized literature, even if the communities here are a huge reservoir of biodiversity. To date, there has been very little research on biomes from Antarctica for the discovery of new photoprotection products, due to the limited accessibility to those ecosystems. Anti-UV molecules as those found, up to date, in Antarctic organisms are found in other latitudes, and have been commercialized. The discovery of new sources of natural sunscreens is highly timely for cosmeceuticals, as many sunscreen bioactives used by humans, such as the widely used octocrylene, imply risks for marine ecosystems. Octocrylene, an ester formed by the reaction of 3,3-diphenylcyanoacrylate with 2-ethylhexanol, has been already found in the liver of dolphins off the coast of Brazil [389], suggesting a potential risk also for humans.

MAAs are the best understood and the most common photoprotective molecules found in marine organisms. Detection of MAAs in fossils supports their protection function against the harmful effects of UV-R in the early geological eras [390]. Thanks to their multiple roles, MAAs are attractive molecules, showing a promising application in the pharmaceutical and cosmetic industries as natural sunscreens, activators of cell proliferation, anti-cancer agents, anti-photoaging molecules, and stimulators of skin renewal [391]. Indeed, several candidates have been tested for sunscreen suitability for potential use in human skin-care and cosmetic products [159,392,393,394,395,396,397,398]. Among these, porphyra-334 from the red algae *Porphyra umbilicalis* associated to shinorine has been commercialized as Helioguard^®^ 365 [394,397,398] with protective properties against UV-A-induced loss of cell viability and DNA damage. Helionori^®^ is another product containing as active ingredients the MAAs sunscreens, palythine, porphyria-334 and shinorine, extracted from *Porphyra umbilicalis*, which protects against UV-A, preserving membrane lipids of keratinocytes and fibroblasts, as well as protecting DNA [399].

The potential application of MAAs with photoprotective and antioxidant activities has been listed in a large number of patents [391]. Besides the application as suncare and cosmetic products, MAAs have been used in the manufacture of several non-biological materials, such as photostabilizing additives in plastics, paints and varnishes [134].

Besides MAAs, also carotenoids have applications in the healthcare and nutraceuticals industry [400]. The global carotenoid market value was $1.5 billion in 2014 and it is expected to increase to nearly $1.8 billion in 2019, with a compound annual growth rate of 3.9% [401]. Microalgae are a valuable source of carotenoids [402] and of some of the most innovative skincare products today. Some examples include Dermochlorella^®^ from CODIF Recherche et Nature (Brittany, France), an extract from green microalgae *Chlorella vulgaris* containing oligopeptides that increases firmness and skin tone [403]; Alguronic acid from Algenist (San Francisco, CA, USA), mix of polysaccharides produced by microalgae with anti-ageing properties; Alguard from FRUTAROM, a natural sulphated polysaccharide from red microalgae *Porphyridium* sp. protecting against skin ageing and photodamage [404]. Analogous sunscreen molecules and photoprotective mechanisms have never been tested from Antarctic species, even if polar sources could potentially yield richer concentrations, or exhibit diverse reaction kinetics [266].

There are only few examples of Antarctic photoprotective molecules that have been already used in biotechnological applications, such as carotenoids isolated from UV-resistant Antarctic bacteria used to develop green solar cells called photosensitizers in Dye Sensitized Solar Cells [297]. Another example is represented by the SeaCode^®^ launched by LIPOTEC, an EPS with a market impact characterized by a mixture of extracellular glycoproteins and other glucidic exopolymers produced by biotechnological fermentation of a *Pseudoalteromonas* sp. isolated in Antarctic waters [405]. This mixture improves the synthesis of collagen I, contributing to the amelioration of skin structural properties [123]. Besides SeaCode^®^, LIPOTEC has developed another anti-aging product named Antarcticine^®^ obtained from extracts of marine *Pseudoalteromonas antarctica* [406].

Currently, there is a need for extensive work to understand the photobiology of Antarctic communities and to investigate on the potential of photoprotective compounds and related molecular and enzymatic machineries from those marine habitats to be used in biotechnological discovery pipelines and pharmaceutical applications. Further development of marine biotechnology for human photoprotection and research must be specially focused on the analysis, biosynthesis, and mode of action of several unknown photoprotective compounds against several abnormalities induced by UV-R. All these studies should be always carried out respecting the environment, and protecting biodiversity and chemical diversity for future generations.

## Abbreviations

ACC	Antarctic Circumpolar Current
ACCE	Antarctic Climate Change and the Environment
APF	Antarctic Polar Front
APX	Ascorbate peroxidase
CAT	Catalase
CDOM	Coloured dissolved organic matter
CFCs	Chlorofluorocarbons
Chl	Chlorophyll
CPDs	Cyclobutane pyrimidine dimers
DOM	Dissolved organic matter
EPS	Extracellular polysaccharides
GHG	Greenhouse gases
GSH	Glutathione
GSH-Px	Glutathione peroxidase
HPLC	High pressure liquid chromatography
MAAs	Mycosporine-like amino acids
MDT	Marine-derived tocopherol
my	Million of years
mya	Million of years ago
NASA	National Aeronautics and Space Administration
NER	Nucleotide excision repair
NOAA	National Oceanic and Atmospheric Administration
ODSs	Ozone depleting substances
PAR	Photosynthetically active radiation
6-4PPs	Pyrimidine 6-4 pyrimidone photoproducts
PUFA	Polyunsaturated fatty acids
ROS	Reactive oxygen species
SOD	Superoxide dismutase
UNEP	United Nations Environment Programme
UV, UV-R	Ultraviolet, UV radiation
WMO	World Meteorological Organization

## Appendix A

**Table A1:** MAAs, UV-absorbing molecules from Antarctic marine organisms.

Taxonomy	Compound	Reference
**Phylum: Heterokontophyta**		
Class: Bacillariophyceae		
*Chaetoceros* sp. 1	Porphyra-334, Shinorine	[152]
*Chaetoceros* sp. 2	Porphyra-334, Shinorine	[152]
*Coretron cryophilum*	Porphyra-334, Shinorine	[213]
*Coscinodiscus centralis*	Porphyra-334, Shinorine	[152]
*Fragilariopsis cylindrus*	Porphyra-334, Shinorine	[152,213]
*Fragilariopsis linearis*	Porphyra-334, Shinorine	[152]
*Porosira glacialis*	Porphyra-334, Shinorine	[152]
*Porosira pseudodenticulata*	Porphyra-334, Shinorine	[152]
*Proboscia inermis*	Porphyra-334, Shinorine	[152]
*Pseudonitzschia sp.*	Porphyra-334, Shinorine	[213]
*Stellarima microtrias*	Porphyra-334, Shinorine	[152]
*Thalassiosira antarctica*	Porphyra-334, Shinorine	[152]
*Thalassiosira tumida*	Porphyra-334, Shinorine	[152]
*Thalassiosira* sp.	Porphyra-334, Shinorine	[213]
Diatom mat (mixture of *Achnantes* sp., *Licmophora* sp., *Navicula* sp.)	Porphyra-334, Shinorine, Palythine	[84]
**Phylum: Chlorophyta**		
Class: Chlorophyceae		
*Enteromorpha bulbosa*	Porphyra-334	[84]
*Monostroma hariotii*	Shinorine	[220]
Green algal mat (mixture of *Ulothrix cf. australis, Urospora* cf. *penicilliformis*)	Porphyra-334, Shinorine, Palythine, Asterina-330	[84]
**Phylum: Ochrophyta**		
Class: Phaeophyceae		
*Desmarestia menziesii*	Porphyra-334	[84]
*Himantothallus grandifolius*	Porphyra-334	[220]
**Phylum: Rhodophyta**		
Class: Rhodophyceae		
*Bangia atropurpurea*	Porphyra-334	[220]
*Curdiea racovitzae*	Porphyra-334, Shinorine, Palythine, Asterina-330, Mycosporine-glycine, Palythene, Palythinol	[84,220]
*Georgiella confluens*	Porphyra-334, Shinorine, Palythine	[220]
*Gigartina skottsbergii*	Porphyra-334, Shinorine, Palythine, Asterina-330	[220]
*Gymnogongrus antarctica*	Shinorine, Palythine, Asterina-330	[220,221]
*Gymnogongrus turquetii*	Porphyra-334, Shinorine, Mycosporine-glycine	[221,222]
*Iridaea cordata*	Shinorine, Palythine, Asterina-330, Mycosporine-glycine, Palythene, Palythinol	[84,219,220]
*Kallymenia antarctica*	Porphyra-334, Shinorine, Palythine, Asterina-330, Mycosporine-glycine, Palythinol	[221,222]
*Lithothamnion* cf. *antarcticum*	Porphyra-334, Shinorine	[84]
*Myriogramme manginii*	Porphyra-334, Shinorine, Palythine, Asterina-330	[220]
*Neuroglossum ligulatum*	Porphyra-334, Shinorine, Palythine	[220,221,222]
*Notophycus fimbriatus*	Porphyra-334, Shinorine, Palythine, Asterina-330, Mycosporine-glycine	[220]
*Pachymenia orbicularis*	Porphyra-334, Palythine, Mycosporine-glycine	[220]
*Palmaria decipiens*	Porphyra-334, Shinorine, Palythine, Asterina-330, Mycosporine-glycine, Palythene, Palythinol, Usujirene	[222]
*Phyllophora antarctica*	Shinorine, Palythine	[219]
*Phyllophora appendiculata*	Shinorine, Palythine, Asterina-330, Mycosporine-glycine, Palythene	[84]
*Plocamium cartilagineum*	Porphyra-334, Shinorine, Palythine, Asterina-330, Palythinol	[220,221]
*Porphyra endiviifolium*	Porphyra-334, Shinorine, Palythine, Asterina-330, Mycosporine-glycine, Palythinol	[222]
*Porphyra plocamiestris*	Porphyra-334, Shinorine	[221]
*Rhodymenia subantarctica*	Porphyra-334	[220]
*Sarcothalia papillosa*	Shinorine, Palythine	[220]
**Phylum: Porifera**		
Class: Demospongia		
*Latrunculia (Latrunculia) apicalis*	Porphyra-334, Shinorine, Palythine, Mycosporine-glycine-valine	[219]
*Cinachyra antarctica*	Porphyra-334, Shinorine, Palythine	[219]
*Tetilla leptoderma*	Porphyra-334, Shinorine, Palythine	[219]
*Haliclona (Gellius) benedeni*	Shinorine, Palythine, Mycosporine-glycine-valine	[219]
*Haliclona* sp.	Palythine, Mycosporine-glycine	[219]
*Homaxinella balfourensis*	Porphyra-334, Shinorine, Palythine, Mycosporine-glycine-valine	[219]
*Inflatella belli*	Palythine	[219]
*Isodictya erinacea*	Palythine, Palythinol	[219]
*Kirkpatrickia variolosa*	Shinorine, Palythine, Mycosporine-glycine-valine	[219]
*Mycale (Oxymycale) acerata*	Palythine	[219]
*Sphaerotylus antarcticus*	Porphyra-334	[219]
*Dendrilla membranosa*	Porphyra-334, Shinorine, Palythine	[219]
*Polymastia invaginata*	Porphyra-334, Shinorine, Palythine	[219]
Unidentified Sponge #1	Porphyra-334, Shinorine, Palythine, Palythene, Palythinol, Mycosporine-glycine, Mycosporine-glycine:valine	[84]
Unidentified Sponge #3	Porphyra-334, Shinorine, Palythine, Mycosporine-glycine	[84]
Unidentified Sponge #5	Porphyra-334, Shinorine, Palythine, Palythene, Mycosporine-glycine, Mycosporine-glycine:valine	[84]
Unidentified Sponge #6	Porphyra-334, Shinorine, Palythine, Mycosporine-glycine, Mycosporine-glycine:valine	[84]
Class: Hexactinellida		
*Rossella nuda*	Palythine	[219]
*Rossella racovitzae*	Palythine, Mycosporine-glycine	[219]
**Phylum: Cnidaria**		
Class: Anthozoa		
*Isotealia antarctica*	Porphyra-334, Shinorine, Palythine, Asterina-330, Mycosporine-glycine, Mycosporine-glycine-valine	[219]
Unidentified Cnidarian #1	Porphyra-334, Shinorine, Palythine, Mycosporine-glycine, Mycosporine-glycine-valine	[219]
**Phylum: Platyhelminthes**		
Class: Rhabditophora		
*Obrimoposthia wandeli*	Porphyra-334, Shinorine, Palythine, Mycosporine-glycine, Mycosporine-glycine-valine	[84]
*Plagiostomum* n. sp.	Porphyra-334, Shinorine, Palythine, Mycosporine-glycine, Mycosporine-glycine-valine	[84]
**Phylum: Nemertea**		
Class: Anopla		
*Parborlasia corrugatus*	Porphyra-334, Shinorine, Palythine, Mycosporine-glycine, Mycosporine-glycine-valine	[84]
*Parborlasia fueguina*	Porphyra-334, Shinorine, Palythine, Mycosporine-glycine, Mycosporine-glycine-valine	[84]
*Amphiporus michaelseni*	Porphyra-334, Shinorine, Palythine, Mycosporine-glycine, Mycosporine-glycine-valine	[84]
**Phylum: Mollusca**		
Class: Polyplacophora		
*Tonicina zschaui*	Porphyra-334, Shinorine, Palythine	[84]
Class: Gastropoda		
*Margarella antarctica*	Porphyra-334, Shinorine, Palythine, Asterina-330	[84]
*Nacella concinna*	Porphyra-334, Shinorine	[407]
*Marseniopsis mollis*	Porphyra-334, Shinorine, Palythine, Mycosporine-glycine	[219]
*Paludestrina antarctica*	Porphyra-334, Shinorine, Palythine, Asterina-330, Palythene, Mycosporine-glycine, Mycosporine-glycine:valine	[84]
*Trophon* cf. *geversianus*	Porphyra-334, Shinorine, Palythine, Asterina-330, Mycosporine-glycine, Palythene, Palythinol	[84]
*Limacina helicina*	Porphyra-334, Shinorine, Palythine, Mycosporine-glycine, Palythenic acid	[135]
*Limacina helicina ssp. antarctica*	Porphyra-334, Shinorine, Palythine, Palythene, Mycosporine-glycine, Mycosporine-glycine:valine	[84]
*Clione antarctica*	Porphyra-334, Shinorine, Palythine, Mycosporine-glycine, Palythenic acid	[135]
*Telarma antarctica*	Porphyra-334, Shinorine, Palythine, Mycosporine-glycine, Mycosporine-glycine:valine	[84]
*Notaeolidia gigas*	Mycosporine-glycine	[219]
*Tritoniella belli*	Shinorine	[219]
	Mycosporine-glycine	[219]
Class: Bivalvia		
*Limatula hodgsoni*	Shinorine, Palythine	[219]
*Limatula* cf. *ovalis*	Porphyra-334, Shinorine, Palythine, Mycosporine-glycine, Mycosporine-glycine:valine	[84]
*Cyamium* cf. *commune*	Porphyra-334, Shinorine, Palythine, Mycosporine-glycine, Mycosporine-glycine:valine	[84]
**Phylum: Anellida**		
Class: Polychaeta		
*Aglaophamus ornatus*	Porphyra-334, Shinorine, Palythine, Palythene, Mycosporine-glycine	[84]
*Neanthes kerguelensis*	Porphyra-334, Shinorine, Palythine, Palythene, Mycosporine-glycine, Mycosporine-glycine:valine	[84]
*Tomopteris carpenteri*	Porphyra-334, Palythine, Mycosporine-glycine	[84]
*Terebella ehlersi*	Porphyra-334, Shinorine, Palythine, Palythene, Asterina-330, Palythinol, Mycosporine-glycine, Mycosporine-glycine:valine	[84]
Unidentified Polychaete #2	Porphyra-334, Shinorine, Palythine, Mycosporine-glycine, Mycosporine-glycine:valine	[84]
Class: Clitellata		
*Trachelobdella autralis*	Porphyra-334, Shinorine, Palythine, Asterina-330, Palythene, Palythinol, Mycosporine-glycine, Mycosporine-glycine:valine	[84]
**Phylum: Artropoda**		
Class: Crustacea		
*Calanus propinquus*	Porphyra-334, Shinorine, Palythine, Palythene, Mycosporine-glycine	[84]
*Euphasia superba*	Porphyra-334, Shinorine, Palythine, Asterina-330, Palythene, Palythinol, Mycosporine-glycine, Mycosporine-glycine:valine, Palythenic acid	[84]
*Cymodocella tubicauda*	Porphyra-334, Shinorine, Palythine, Palythene, Mycosporine-glycine, Mycosporine-glycine:valine	[84]
*Notasellus sarsii*	Porphyra-334, Shinorine, Palythine, Palythene, Mycosporine-glycine, Mycosporine-glycine:valine	[84]
*Glyptonotus antarcticus*	Palythine	[219]
*Bovallia gigantea*	Porphyra-334, Shinorine, Palythine, Mycosporine-glycine, Mycosporine-glycine:valine	[84]
*Halirages* sp.	Porphyra-334, Shinorine, Palythine, Palythene, Mycosporine-glycine, Mycosporine-glycine:valine	[84]
*Jassa* sp.	Porphyra-334, Shinorine, Palythine, Palythene, Mycosporine-glycine, Mycosporine-glycine:valine	[84]
*Orchomene* sp.	Porphyra-334, Shinorine, Palythine, Palythene, Mycosporine-glycine, Mycosporine-glycine:valine	[84]
*Paraceradocus* sp.	Porphyra-334, Shinorine, Palythine, Asterina-330, Palythene, Mycosporine-glycine, Mycosporine-glycine:valine	[84]
*Pariphimedia integricauda*	Porphyra-334, Shinorine, Palythine, Asterina-330, Palythene, Mycosporine-glycine, Mycosporine-glycine:valine, Palythinol	[84]
*Pontogeneia* sp.	Porphyra-334, Shinorine, Palythine, Asterina-330, Palythene, Mycosporine-glycine, Mycosporine-glycine:valine, Palythinol	[84]
Class: Pycnogonida		
*Achelia spicata*	Porphyra-334, Shinorine, Palythine, Palythene, Mycosporine-glycine, Mycosporine-glycine:valine	[84]
Unidentified Pycnogonid	Shinorine, Palythine	[219]
**Phylum: Bryozoa**		
Class: Gymnolaemata		
*Beania livingstonei*	Porphyra-334, Shinorine	[84]
*Inversiula nutrix*	Porphyra-334, Shinorine, Palythine, Palythene, Mycosporine-glycine, Mycosporine-glycine:valine	[84]
**Phylum: Echinodermata**		
Class: Crinoidea		
*Promachocrinus kerguelensis*	Porphyra-334, Shinorine, Palythine, Mycosporine-glycine	[219]
Class: Asteroidea		
*Granaster nutrix*	Porphyra-334, Shinorine, Palythine, Asterina-330, Mycosporine-glycine, Mycosporine-glycine:valine	[84]
*Odontaster validus*	Palythine	[219]
	Mycosporine-glycine	[219]
Class: Echinoidea		
*Sterechinus neumayeri*	Porphyra-334, Shinorine, Palythine, Mycosporine-glycine	[191,219]
Class: Holothuroidea		
*Cucumaria ferrari*	Porphyra-334, Shinorine, Palythine, Mycosporine-glycine, Mycosporine-glycine:valine	[219]
*Ekmocucumis steineni*	Porphyra-334, Palythine, Mycosporine-glycine, Mycosporine-glycine:valine	[84]
*Amphioplus affinis*	Palythine	[84]
**Phylum: Chordata**		
Class: Ascidiacea		
*Cnemidocarpa verrucosa*	Porphyra-334, Shinorine, Palythine	[219]
*Molgula enodis*	Porphyra-334, Shinorine, Palythine, Asterina-330, Mycosporine-glycine, Mycosporine-glycine:valine, Palythene	[84]
Unidentified ascidia	Shinorine, Mycosporine-glycine	[219]
Class: Actinopterygii		
Unidentified ice-fish larvae	Porphyra-334, Shinorine, Palythine, Mycosporine-glycine, Mycosporine-glycine:valine, Palythene	[84]
*Trematomus bernacchii*	Shinorine, palythine	[219]

**Table A2:** UV-absorbing molecules, that are not MAAs, from Antarctic marine organisms.

Taxonomy	Compound	Reference
**Phylum: Porifera**		
Class: Demospongia		
*Isodictya erinacea*	Erebusinone	[228]
**Phylum: Proteobacteria**		
Class: Gammaproteobacteria		
*Alteromonas stellipolaris* sp. nov.	Melanin	[241]
**Phylum: Rhodophyta**		
Class: Florideophyceae		
*Palmaria decipiens*	UV-adsorbing pigment	[245]
**Phylum: Chlorophyta**		
Class: Ulvophyceae		
*Enteromorpha bulbosa*	UV-adsorbing pigment	[245]
**Phylum: Ochrophyta**		
Class: Phaeophyceae		
*Adenocystis utricularis*	Phlorotannins	[266]
*Ascoseira mirabilis*	Phlorotannins	[266]
*Chordaria linearis*	Phlorotannins	[266]
*Cystosphaera jacquinotii*	Phlorotannins	[266]
*Desmarestia anceps*	Phlorotannins	[266,408,409]
*Desmarestia antarctica*	Phlorotannins	[266]
*Desmarestia menziesii*	Phlorotannins	[266,408,409]
*Himantothallus grandifolius*	Phlorotannins	[266]
*Phaeurus antarcticus*	Phlorotannins	[266]

**Table A3:** Antioxidant compounds from Antarctic marine organisms.

Taxonomy	Compound	Reference
**Phylum: Bacteroidetes**		
Class: Flavobacteria		
*Antarcticimonas flava*	Carotenoids	[298]
*Muricauda antarctica*	Carotenoids	[299]
**Phylum: Proteobacteria**		
Class: Gammaproteobacteria		
*Pseudoalteromonas haloplanktis* TAC125	Enzymatic antioxidant defence	[338]
**Phylum: Cyanobacteria**		
Class: Hormogoneae		
*Nostoc* sp.	Carotenoids	[294]
*Nostoc* sp.	Carotenoids	[295]
*Nostoc commune*	Carotenoids	[296]
*Nostoc* sp.	Carotenoids	[301]
Class: Cyanophyceae		
*Anabaena* sp.	Carotenoids	[301]
*Phormidium* sp.	Carotenoids	[301]
*Oscillatoria* sp.	Carotenoids	[295]
**Phylum: Bacillariophyta**		
Class: Mediophyceae		
*Thalassiosira antarctica*	Diadinoxantin, diatoxanthin	[293]
*Thalassiosira weissflogii*	Diadinoxantin, diatoxanthin	[293]
*Thalassiosira* sp.	α-tocopherol	[312]
*Chaetoceros brevis*	SOD	[331]
**Phylum: Euglenozoa**		
Class: Euglenophyceae		
*Eutreptiella* sp.	Xanthophylls	[292]
**Phylum: Miozoa**		
Class: Dinophyceae		
*Polarella glacialis*	Xanthophylls	[291]
**Phylum: Chlorophyta**		
Class: Ulvophyceae		
*Enteromorpha bulbosa*	UV-absorbing pigments	[245]
Class: Trebouxiophyceae		
*Chlorella* sp.	Ascorbic acid	[322]
Class: Chlorophyceae		
*Chlamydomonas sp.* ICE-L	SOD, CAT, peroxidase	[105]
**Phylum: Rhodophyta**		
Class: Florideophyceae		
*Palmaria decipiens*	UV-absorbing pigments	[245]
*Leptosomia simplex*	Carotenoids	[410]
*Delesseria lancifolia*	Violaxanthin, antheraxanthin, zeaxanthin	[302]
**Phylum: Cnidaria**		
Class: Hydrozoa		
*Errina antarctica*	Astaxanthin	[307]
**Phylum: Mollusca**		
Class: Bivalvia		
*Laternula elliptica*	α-tocopherol	[313]
	Enzymatic antioxidant defence	[332]
**Phylum: Arthropoda**		
Class: Malacostraca		
*Euphausia superba*	Astaxantin	[217]
	Astaxanthin	[305]
	Vitamin A	[305]
	Vitamin E	[305]
	α-tocomonoenol	[317]
**Phylum: Chordata**		
Class: Chondrichthyes		
*Raja georgiana*	Carotenoids	[306]
Class: Actinopterygii		
*Muraenolepis microps*	Carotenoids	[306]
*Dissostichus eleginoides*	Carotenoids	[306]
*Notothenia gibberifrons*	Carotenoids	[306]
*Notothenia rossi-marmorata*	Carotenoids	[306]
*Trematomus hansoni*	Carotenoids	[306]
*Chaenocephalus aceratus*	Carotenoids	[306]
*Champsocephalus gunnari*	Carotenoids	[306]
*Pseudochaenichthys georgianus*	Carotenoids	[306]
*Pagothenia borchgrevinki*	Vitamin E	[316]
*Trematomus bernacchii*	Vitamin E	[316]
*Champsocephalus gunnari*	α-tocomonoenol	[317]
*Gobionotothen gibberifrons*	α-tocomonoenol	[317]
*Chaenocephalus aceratus*	α-tocomonoenol	[317]
Antarctic notothenioid fishes	Enzymatic antioxidant defence	[333,334,335,336,337]

**Table A4:** Molecules involved in the mechanisms of repair in Antarctic marine organisms.

Taxonomy	Compound	Activity	Reference
**Phylum: Proteobacteria, Bacteroidetes**			
Class: Alphaproteobacteria, Betaproteobacteria, Gammaproteobacteria, Flavobacteriia			
*Pseudomonas, Janthinobacterium, Flavobacterium, Hymenobacter, Sphingomonas*	Photolyase	DNA repair	[3,355]
**Phylum: Ciliophora**			
Class: Spirotrichea			
*Euplotes focardii*	Heat shock protein, Hsp70	Chaperone in the UV stress	[382]
**Phylum: Chlorophyta**			
Class: Chlorophyceae			
*Chlamydomonas sp.* ICE-L	Photolyase	DNA repair	[356]
	Heat shock protein, Hsp70	Chaperone in the UV stress	[381]
**Phylum: Ochrophyta**			
Class: Bacillariophyceae			
*Chaetoceros neglectus, Corethron cryophilum, Coscinodiscus ocutus-iridis, Odontella weissflogii, Porosira pseudodenticulata, Thalassiosira subtilis, Eucampia antarctica, Chaetoceros convolutus, Chaetocerois socialis, Thalassiosira australis, Licmophora decora, Nitzchia kerguelensis*	Photolyase/NER	DNA repair	[96]
**Phylum: Echinodermata**			
Class: Echinoidea			
*Sterechinus neumayeri*	Photolyase	DNA repair	[359]
**Phylum: Arthropoda**			
Class: Crustacea			
*Euphausia superba*	Photolyase	DNA repair	[103]
**Phylum: Chordata**			
Class: Actinopterygii			
*Chaenocephalus aceratus, Notothenia coriiceps*	Photolyase	DNA repair	[103]
**Organismal assemblages**			
Antarctic marine bacterioplankton communities	RecA protein	DNA repair	[367]
Antarctic phytoplankton communities	Photosystem II (PSII) repair cycle	*de novo* synthesis	[368]
